# Validation of a Method Scope Extension for Simple Biomonitoring of 353 Pollutants in Serum Samples

**DOI:** 10.3390/toxics11060498

**Published:** 2023-05-31

**Authors:** Cristian Rial-Berriel, Álvaro Ramos-Luzardo, Andrea Acosta-Dacal, Ana Macías-Montes, Pilar Fernández-Valerón, Luis Alberto Henríquez-Hernández, Manuel Zumbado, Luis D. Boada, Octavio P. Luzardo

**Affiliations:** 1 Toxicology Unit, Research Institute of Biomedical and Health Sciences (IUIBS), Universidad de Las Palmas de Gran Canaria, Paseo Blas Cabrera s/n, 35016 Las Palmas de Gran Canaria, Spain; alvaro.ramos@ulpgc.es (Á.R.-L.); andrea.acosta@ulpgc.es (A.A.-D.); ana.macias@ulpgc.es (A.M.-M.); pilarfdez.valeron@ulpgc.es (P.F.-V.); luis.henriquez@ulpgc.es (L.A.H.-H.); manuel.zumbado@ulpgc.es (M.Z.); luis.boada@ulpgc.es (L.D.B.); octavio.perez@ulpgc.es (O.P.L.); 2 Spanish Biomedical Research Center in Physiopathology of Obesity and Nutrition (CIBERObN), Instituto de Salud Carlos III, 28029 Madrid, Spain

**Keywords:** serum, POPs, pesticides, biomonitoring, pharmaceuticals

## Abstract

Animals and humans are exposed to various residues that can have a detrimental impact on health, including carcinogenic potential, endocrine disruption, or fatal toxicity. The toxic burden can be evaluated in several biological samples, with serum being one of the preferred and most convenient options. In this study, we have applied and validated a method for detecting several hundred toxins in serum samples. This technique involved a single-step QuEChERS (quick, easy, cheap, effective, rugged, and safe) extraction followed by analysis using gas and liquid chromatography coupled with mass spectrometry. With this methodology, we could detect and quantify up to 353 compounds, including persistent organic pollutants (POPs), pesticides, pharmaceuticals, and rodenticides, using just 250 µL of serum. Among them, 92% could be measured at concentrations below 1.25 ng/mL, making it ideal for biomonitoring. We applied this method to samples collected from camels (*n* = 40) and humans (*n* = 25). We detected naproxen, ketoprofen, paracetamol, levamisole, and some POPs in these samples. This study validated the ability to simultaneously detect a broad range of compounds in small volumes of serum.

## 1. Introduction

Environmental pollution is poised to be one of the most significant challenges of the upcoming years, and its sources vary depending on the compounds involved. There exist thousands of toxic chemicals, including pesticides widely used in agriculture and farming, medicines such as nonsteroidal anti-inflammatory drugs (NSAIDs), antibiotics, and persistent organic pollutants (POPs). Some examples of POPs include polychlorinated biphenyls (PCBs), polybrominated diphenyl ethers (PBDEs), polycyclic aromatic hydrocarbons (PAHs), organochlorine pesticides (OCPs), and new flame retardants (NFRs), among others [1,2]. The compounds referred to as PAHs are created as byproducts of industrial processes, while PCBs and PBDEs are intentionally manufactured for use in industrial applications, specifically as thermal and electrical insulation. On the other hand, OCPs, such as DDT, are utilized as powerful pesticides due to their toxicity. These substances share the common characteristics of being highly persistent in the environment and having the ability to accumulate in living organisms, which can result in developmental or neurological issues, disruption of the endocrine system, and the development of cancer [2,3,4]. Due to their persistence, these compounds are still commonly detected in both living organisms and the environment, despite many of them having been banned several decades ago [5].

On the other hand, the overuse of semipersistent pesticides, including carbamates, pyrethroids, neonicotinoids, and organophosphates, particularly in agriculture to meet the demands for high food and feed production, can result in exposure to these chemicals, primarily through food consumption. These pesticides are known to be endocrine disruptors, carcinogens and pose significant environmental hazards to beneficial insects [6,7,8,9,10]. Similarly, in both human and veterinary medicine, the excessive use of medications, such as antibiotics and NSAIDs, can result in the presence of their residues in food, either directly or indirectly through water contamination, such as sewage sludge or seawater. [11,12]. Apart from their potential toxicity to living organisms (for example, acetaminophen is hepatotoxic, salicylic acid can cause haematological disorders, and some antibacterial and antifungal compounds are nephrotoxic), the use of antibiotics can also contribute to the emergence and spread of bacterial resistance, which is considered one of the most significant threats to human health [13,14,15].

Due to the significant environmental impact of these chemicals, biomonitoring is a critical tool to assess the toxic burden on the body [16,17,18]. For biomonitoring studies, the preferred sample types include whole blood, umbilical cord blood, urine, or breast milk [1,19,20,21]. However, serum is the preferred choice because is easily obtained and can be used to study the entire population, regardless of age or gender, unlike breast milk [1,22]. Compared to whole blood, serum offers several advantages in biomonitoring studies. It is a simpler and more homogeneous matrix, containing no fibrin or erythrocytes, fewer proteins (which may interfere with ionization in the detection of these substances [23,24]), and higher levels of sodium and potassium [16,19].

Therefore, it is essential to have access to straightforward, accurate, and reliable multicompound analytical methods that comply with the standards established by international analytical agencies [25,26] and work with small sample volumes [12]. To achieve this, the extraction of analytes from the matrix and subsequent detection and quantification are required. The most-used extraction methods for serum analysis are solid-phase extraction (SPE), liquid–liquid extraction (LLE), and, more recently, solid-phase microextraction (SPME) [3,6,21,27]. In recent years, QuEChERS (quick, easy, cheap, effective, rugged, and safe) based extraction methods have been developed and validated, as they allow for customization and optimization for specific purposes [9,16,17,18,28]. This type of extraction can handle the processing of large numbers of samples using a simple methodology, unlike SPE or LLE, which commonly require a larger volume of solvents that may be more harmful to the environment [10,21] or involve additional steps such as pH adjustment [1,11,13,14], making them more time-consuming and expensive [6,16,27,29].

Different types of detectors and chromatography are employed depending on the intended use. Most of the recently optimized methods utilize liquid chromatography (LC) and gas chromatography (GC) coupled to a mass spectrometer (MS/MS), high-resolution mass spectrometry (HRMS/QTOF), or flame ionization detector (FID) [3,13,19,28,30]. These detection and quantification methods are recommended and the most frequently used to cover a broad range of analyte groups with varying physicochemical characteristics in targeted analyses [21,25,28].

The objective of this study was to expand and validate a versatile QuEChERS and LC- and GC-MS/MS method that had previously been optimized for whole blood. This method was used for analysing a variety of substances, including persistent organic pollutants (POPs), semipersistent pesticides like rodenticides, carbamates, pyrethroids, and neonicotinoids, as well as antibiotics, antifungal agents, and nonsteroidal anti-inflammatory drugs (NSAIDs) used for human and animal medicines. Finally, we applied this method to two sets of camel and human serum samples to verify its usefulness in real-world scenarios.

## 2. Materials and Methods

### 2.1. Chemicals and Solutions

The solvents involved were MS/MS grade acetonitrile (ACN), formic acid (FA), and methanol (MeOH), provided by Honeywell (Morristown, NJ, USA). Water (18.2 MΩ/cm) was purified daily in the laboratory using a Millipore MiliQ A10 Gradient system (Molsheim, France). Foetal bovine serum (FBS) provided by ThermoFisher (Waltham, MA, USA) was used as a blank matrix (tested before the start of validation experiments), till real serum could be quite heterogeneous, with detectable quantities of our analytes of interest.

Premixed QuEChERS salts for the AOAC (Association of Official Agricultural Chemists) method (1.5 g of sodium acetate and 6 g of magnesium sulphate) were purchased from Agilent Technologies (Palo Alto, CA, USA). Ammonium acetate (MS/MS grade, for LC mobile phases) was purchased from Fisher Scientific (Loughborough, UK).

MS/MS grade standards (93 to 99% purity) for all the analytes and deuterated compounds included were obtained from European Pharmacopoeia Reference Standards (Strasbourg, France), CPA Chem (Stara Zagora, Bulgaria), A2S—Analytical Standard Solutions (Saint Jean D’Illac, France), Dr. Ehrenstorfer (Augsburg, Germany), and Sigma-Aldrich (Augsburg, Germany), according to availability, purity, and presentation (solid/liquid, preferably solid for its greater stability).

Three independent working solutions (one for pesticides (diluted in ACN), another for pharmaceuticals (in ACN), and one for POPs (in acetone)) at 1 ug/mL each were prepared for the validation experiments. A procedural internal standard (P-IS) mix composed of 11 deuterated compounds (acenaphthene-d10, atrazine-d5, carbendazim-d3, chlorpyrifos-d10, chrysene-d12, cyromazine-d4, diazinon-d10, linuron-d3, PCB 200, pirimicarb-d6, and phenanthrene-d10) were prepared at 1 µg/mL and added to all samples before extraction to check for whole-method errors. These working solutions were kept at −20 °C for not more than 1 year and checked before each use.

### 2.2. Instrumental Analysis

As we published previously, two consecutive analyses in LC- and GC-MS/MS are needed to achieve the scope of the method. Since this method is an extension of an existing procedure [17,31], the chromatographic and mass-spectrometry parameters will be presented in a summarised form [17,31]. MassHunter Quantitative and Qualitative software were used for data acquisition and analysis.

#### 2.2.1. UHPLC-MS/MS

Eight microlitres of the final extract were injected at a flow of 0.4 mL/min in an ultrahigh-performance liquid chromatograph (UHPLC), model 1290 Infinity II, coupled to a triple quadrupole mass spectrometer (MS/MS) model 6460 with positive/negative switching mode, obtained from Agilent Technologies (Palo Alto, CA, USA). Since we used no cleanup step, an online filter and a guard precolumn to retain particles were coupled to the analytical column (InfinityLab Poroshell 120). Mobile phases consisted of (A) 2 mM ammonium acetate, 0.1% FA in MiliQ water and (B) 2 mM acetate in MeOH. Mobile phase B gradient was started at 5% (up to 0.5 min), followed by 20% at 1 min, 40% at 2.5 min, 85% at 8 min and 100% from 10 min to 14 min. The total running time was 18 min. Nitrogen (99.9999%) was used as the collision gas. The temperatures, flows, and pressures of chromatographic and mass spectrometer were the same as the original method [17,31]. Optimized retention time (RT) and transitions are shown in Table 1.

#### 2.2.2. GC-MS/MS

The analysis of most nonpolar compounds involved the use of an Agilent Gas Chromatograph 7890B connected to a Triple Quad 7010 mass spectrometer equipped with Electron Impact ionization, manufactured by Agilent Technologies in Palo Alto, CA. To facilitate the analysis, two columns with a length of 15 m each, a diameter of 0.25 mm, and a film thickness of 0.25 µm, J&W HP-5MS from Agilent Technologies, were joined by an ultimate purged union, which enabled the application of back flush after each analysis. The oven temperature was increased according to a specific ramp protocol, starting at 80 °C for 1.8 min and then increasing at a rate of 40 °C per minute until reaching 170 °C. The temperature was then raised by 10 °C per minute until it reached 310 °C, where it was maintained for 3 min, resulting in a total run time of 21.05 min. The carrier gas used was helium (99.999%) which was kept at a constant flow rate of 1 mL/min, while nitrogen (99.9999%) was used as a collision gas (supplied by Linde, Dublin, Ireland). An aliquot of 1.5 µL was injected through an ultrainert glass–wool inlet liner, in spitless mode. Details of the inlet, backflush, and source parameters have been previously published [17,31]. Transitions and retention time are shown in Table 1.

### 2.3. Sample Preparation and Extraction Procedure

The extraction method is a modification of the original technique proposed by Anastassiades et al. in 2003 [32]. Since its invention, numerous modifications have been made to adapt it to new matrices [33]. The present study is a scope extension of a method previously presented by our group on whole-blood matrix [17,31]. In brief, a 2 mL tube was used to add P-IS mix to 250 µL of homogenized serum, which was then vortexed for 30 s. These samples were then fortified to be used for the calibration curve, quality control (QC) samples, and validation experiments. Afterward, the samples were left on an orbital shaker for 1 h to ensure proper mixing and equilibration between the matrix components and analytes. Next, 500 µL of acidified acetonitrile (ACN) containing 1% formic acid (FA) was added. This step results in the precipitation of proteins, which enables higher extraction efficiency for the compounds at acidic pH [11]. Subsequently, the samples were thoroughly mixed and subjected to ultrasonic treatment for 20 min to promote contact between the analytes and the solvent and to facilitate the separation of analytes from the matrix. The samples were then microcentrifuged for 10 min at −2 °C and 4200 RPM (RCF = 1992× *g*, radius 101 mm) to facilitate lipid coagulation and sedimentation. The resulting supernatant was filtered through a 0.2 um pore filter manufactured by Macherey-Nagel in Düren, Germany and collected in a vial with an insert suitable for injection into LC and GC systems. It is important to note that all samples and QC samples were subjected to the same extraction method.

### 2.4. Validation Experiments/Procedures

Validation assays were performed in accordance with two analytical guides: the Scientist Working Group for Forensic Toxicology (SWGTOX) [26] and the European guide for pesticide analysis in food and feed (SANTE) [25]. The validation parameters included identity (qualifier ratio, retention time, signal-to-noise ratio, and peak shape), selectivity (determination of interferences in blank matrix samples at each analyte retention time), linearity (determination of working range), accuracy (bias and precision), carryover, limit of quantification (LOQ), uncertainty (based on intralaboratory validation/data), and matrix effect (ME). For linearity, a 12-point matrix-matched calibration curve was prepared for each experiment, covering a range of 0.1 to 40 ng/mL. Precision and accuracy were assessed in quintuplicate using relative standard deviation (RSD) and bias, respectively. LOQ was determined as the lowest concentration meeting the criteria for identity, accuracy, and precision. Carryover was evaluated by injecting a blank sample after a sample fortified at 50 ng/mL. Uncertainty was calculated following the 1st approach of SANTE 2021, based on bias and precision, from intralaboratory QC data [25] with an expanded coverage factor k = 2. Finally, ME was assessed by comparing responses between an extract of blank matrix (fortified at least three levels in triplicate) and spiked acetonitrile.

### 2.5. Applicability of the Method

Two sets of serum samples were selected to assess the method’s applicability for detecting 353 analytes: one from human patients (*n* = 25) at the Complejo Hospitalario Materno-Insular in Las Palmas de Gran Canaria, Spain, and the other from camels (*n* = 40) in a tourist excursion herd in the dunes of Maspalomas in the south of Gran Canaria. Human serum samples were collected after obtaining informed consent from the patients and approved by Drug Research Ethics Committee (CEIm code: 2022-266-1). Serum samples were obtained by centrifuging whole blood without anticoagulants. Camel serum samples were obtained by venipuncture and were received in the laboratory ready for processing, having been refrigerated.

## 3. Results and Discussion

### 3.1. Optimization of Extraction, Separation and Detection

Numerous methods exist for detecting and measuring various substances but they typically target specific analytes within a particular group, such as POPs [16,34], pesticides [9,19], or pharmaceuticals [30]. These methods involve SPE, LLE, or derivatization processes, which can make analysis time-consuming and complex. To our knowledge, this study is the first that involves one-step extraction and quantitative detection of a large number of compounds with diverse physicochemical properties, based on QuEChERs extraction for serum samples.

In this study, we validated a pre-existing method that was initially designed for whole blood, for use in serum [17,31]. Even slight differences between these two matrices can significantly impact the extraction and quantification of certain compounds due to potential interferences with proteins, fats, and other molecules that may affect the detection process. Therefore, a thorough validation process was necessary for this modified method. The method allows for the quantitative detection of 353 compounds in serum, including 56 POPs, 10 anticoagulant rodenticides, 233 pesticides, and 54 pharmaceuticals, without any changes to the mobile phase, oven ramp, or other chromatographic parameters. Only the retention times, qualifier ratios, and qualifier/quantifier transitions were adjusted for technical reasons. Table 1 provides a complete list of compounds and summarizes the chromatographic and spectrometer parameters used in the study.

As mentioned in Section 2.3, we opted not to perform a cleanup step (such as PSA, C18, EMR-lipid) to prevent any loss of analytes [19,25]. Furthermore, this method saves both material and time while still ensuring the effectiveness and durability of the equipment. It allows for the analysis of 353 compounds while complying with the criteria parameters established in international analytical guides such as SANTE and SWGTOX [25,26]. The validation parameters studied include selectivity, linearity, accuracy, precision, LOQ, uncertainty, carryover, and matrix effect.

### 3.2. Validation Experiments

**Figure 1:**
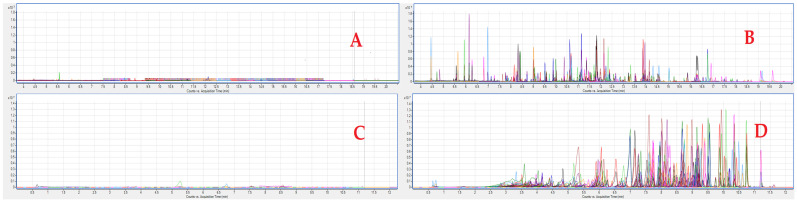
GC- and LC-MS/MS chromatograms of blank and fortified (at 40 ng/mL) serum. (**A**) GC blank; (**B**) GC fortified; (**C**) LC blank; and (**D**) LC fortified. The color lines correspond to the chromatogram of each compound.

For this analytical validation work, of the 353 compounds covered in this work, 129 were analyzed by GC and 224 by LC, from a single QuEChERS extraction. Compared to the previously validated whole-blood method, which was able to detect 360 substances, six analytes were found to meet validation criteria in serum but not in whole blood (four pesticides: dichlorvos, metalaxyl, methiocarb-sulfone, pirimicarb-desmethyl, and two pharmaceuticals: moxidectin and sulfapiridine). Conversely, thirteen compounds met the same validation criteria in whole blood but not in serum (five pesticides: carbosulfan, methomyl oxime, nitenpyram, paraoxon methyl, and parathion ethyl, and eight pharmaceuticals: cloxacillin, dicloxacillin, marbofloxacin, nafcillin, penicillin g, piperacillin, sarafloxacin, and sulfapyridine). All 56 COPs and 10 anticoagulant rodenticides were validated with similar LOQs. To verify the selectivity of the method, a blank matrix was analyzed and no interferences were detected at concentrations near the LOQ (Figure 1).

Table 1 presents the regression coefficients (R^2^) achieved during the linearity assessment for each analyte within the working range (minimum of five levels, ranging from LOQ to 40 ng/mL). Due to the broad-spectrum and multiresidue nature of the method, which targets several hundred substances from diverse groups, not all compounds attained ideal regression coefficients of 0.999. The lowest coefficient value was obtained for endosulfan sulfate (0.9251). For formetanate, enrofloxacin, diphacinone, p,p′-DDT, benfuracarb, chlorophacinone, phenylbutazone, and danofloxacin, the R^2^ was below 0.95. However, for the remaining compounds (92% of the total list), the R^2^ was >0.97, comparable to the linearity outcomes of whole blood, where 95% of compounds showed R^2^ > 0.95.

To test the recovery and precision, at least five levels were examined in quintuplicate measurements for all 353 compounds included in the method, comparing the spiked and extracted FBS response with the spiked blank extracts. Both inter- and intraday precision were calculated as % RSD. According to the guidelines, satisfactory recovery ranges from 70–120% with RSD < 20% [25]. The recoveries ranged from 70.85 to 126.43, with RSD ranging from 0.28 to 24.98% (Appendix A). Despite not strictly meeting the criteria mentioned above, certain important compounds for poison diagnostic studies and biomonitoring were included, such as flusilazole (recovery 126%, intraday precision 13.07%) and sulfamonomethoxine (120%, 11.58%, respectively). When RSDs are low, accuracy (60–140%) exceptions are permitted [25]. Following SANTE’s recommendation, expanded uncertainties were calculated using the first approach in Appendix C of the SANTE guide [25]. All MUs were below 60.01% (individual MUs for each compound were shown in Appendix A.

To determine the LOQs for each chemical, recoveries and precision were calculated in quintuplicate at low concentrations of the curve. The lowest concentration that could be quantified with acceptable accuracy and precision was considered the LOQ for each compound. All compounds had LOQs below 5 ng/mL, and up to 92.35% of the substances included in the method could be quantified at concentrations below 1.25 ng/mL, as shown in Table 1. This is comparable to the previously published method for whole blood, where up to 95% of the compounds were detected below 1.5 ng/mL [17,31], as well as to other multiresidue studies for pesticides [19] and POPs [27] in serum.

The presence of a high concentration of analyte in a sample that remains on the column and interferes with the quantification of subsequent injections is known as carryover. To test for carryover, FBS fortified to 50 ng/mL was injected, followed by a blank. Carryover was considered present if quantification exceeded 10% of the LOQ. No carryover effect was observed for any of the 353 compounds.

**Figure 2:**
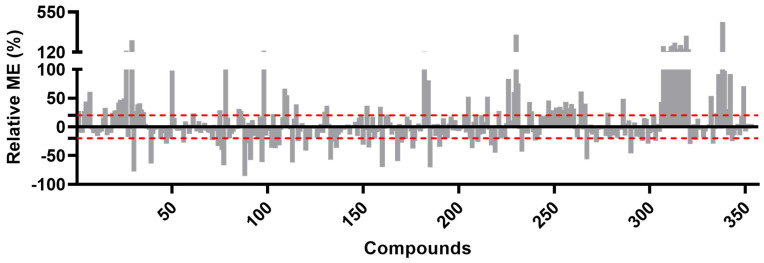
Relative Matrix Effect (%) of 353 compounds (the red dotted line indicates the acceptable range, from −20% to +20%).

Serum or blood are biological matrices that are known to be complex and contain various components such as lipids, proteins, pigments, and cellular debris, which can vary between seemingly similar matrices [35]. Serum is considered a more homogeneous sample with a lower matrix burden compared to other biological matrices such as blood, which contain various components such as lipids, proteins, pigments, and cellular debris that can differ even in similar matrices [9]. Matrix components can interfere with compound ionization and significantly affect quantification, necessitating evaluation of ME. This was done by testing three identified concentrations (2, 10, and 20 ng/mL, each triplicate) in spiked serum extracts, quantified using a calibration curve made with ACN. ME was observed in both liquid and gas chromatography techniques (Table 1), ranging from 14.81 to 543.45%. Among the 353 compounds, 206 (58.35%) had acceptable ME within 80–120% range according to the SANTE analytical guide [25]. On the other hand, it was observed that 90 out of 353 compounds (25.5%) showed signal enhancement, while 57 (16.15%) exhibited signal suppression due to matrix components (as shown in Figure 2). Furthermore, in the present study, a greater number of compounds underwent matrix effects in LC compared to GC (61% versus 53.6%, respectively), which is in line with previous findings [16,17,25]. In conclusion, a matrix-matched calibration was selected as the preferred method of calibration, which was recommended by the SANTE analytical guide [25,26]. This choice was supported by the ME results, which showed that over 41% of the compounds had medium or strong ME.

Given its ability to monitor hundreds of compounds, including POPs, nonbanned pesticides such as anticoagulant rodenticides and commonly used pharmaceuticals, the present method appears to be a valuable option for biomonitoring purposes. Furthermore, it can be applied to cases involving pesticide and pharmaceutical poisoning or overdose.

### 3.3. Application to Real Samples

The method developed was utilized to analyze two sets of actual serum samples: one from humans (*n* = 25) and the other from camels (*n* = 40) to validate its suitability for the intended purpose. Appendix B provides a summary of the identified/quantified compounds. No nonbanned pesticides were found in either series. This result was expected, given the existing legislation that establishes maximum residue levels (MRLs) for food and feed, which implies that exposure to anticoagulant rodenticides and pesticides was not anticipated. Likewise, although rodenticides are prevalent in food chains, anticoagulant rodenticides were absent in both humans and camels. However, while camels were free of the analysed toxics, measurable concentrations of pharmaceuticals and some POPs were found in human serum samples. The camels were young, had not received any pharmacological treatments, were herbivorous, and were fed commercial feed, which was subject to stringent controls.

In contrast, human serums showed detection of six POPs above LOQs, including PCB congeners 138, 153, 180, p,p′-DDE, hexachlorobenzene (HCB), hexachlorocyclohexane beta (β-HCH), and naphthalene (Appendix B). These findings were consistent with earlier research in the Canary Islands where the most commonly identified compounds were p,p′-DDE, HCB, β-HCH, naphthalene, and PCB congeners 138, 153, and 180 [2,34].

In the end, four pharmaceuticals (acetaminophen, ketoprofen, naproxen, and levamisole) were detected in nine (36%), three (12%), three (12%), and two (8%) human samples, respectively. Among them, naproxen, ketoprofen, and acetaminophen are commonly used NSAIDs for humans, with maximum concentrations of 4058.04, 539.64, and 30.93 ng/mL, respectively, which suggests recent pharmaceutical use. Interestingly, levamisole was unexpectedly present in two human samples. Levamisole is an antiparasitic used in veterinary medicine that is frequently used as a cocaine adulterant, implying that exposure to this pharmaceutical could be related to cocaine inhalation, posing a potential health risk, not only due to cocaine but also because levamisole can cause agranulocytosis in humans [36].

## 4. Conclusions

The described approach is an expansion of an analytical technique used to identify 353 compounds (encompassing 56 POPs, 233 pesticides, 10 rodenticides, and 54 pharmaceuticals) in 250 µL of serum. The technique utilizes QuEChERS extraction, quantification with LC and GC-MS/MS, and conforms to international analytical standards. The proposed method enables the determination of 95% of compounds at levels below 1.25 ng/mL, with reliable reproducibility and recoveries, making it suitable for the analysis of trace residues. Its applicability to authentic samples confirms its usefulness for biomonitoring studies and diagnostic applications in cases of poisoning.

## Figures and Tables

**Table 1:** Summary of compounds (including compound class) and chromatographic and mass spectrometric conditions of analytes in serum, linearity, matrix effect (ME), and limit of quantification (LOQ).

No.	Compound	Class	Technique	Retention Time (min)	Polarity	Quantification		Confirmation		Fragmentor Voltage (V)	Linearity (R^2^)	ME (%)	LOQ (ng/mL)
						MRM (m/z)	Collision Energy (eV)	MRM (m/z)	Collision Energy (eV)				
1	2-Phenylphenol	P	GC	6.28	positive	169.0 ➔ 115.0	30	169.0 ➔ 141.0	15	70	0.9802	127.84	0.6
2	4,4′-Dichlorobenzophenone (metabolite of dicofol)	P	GC	10	positive	250.0 ➔ 139.0	15	250.0 ➔ 215.0	5	70	0.9762	90.4	0.3
3	Abamectine	P	LC	10.98	positive	890.5 ➔ 567.1	10	895.5 ➔ 751.4	45	160	0.9811	89.99	2.5
4	Acenaphthene	POP	GC	5.93	positive	153.0 ➔ 152.0	25	153.0 ➔ 151.0	35	70	0.9846	127.45	0.3
5	Acenaphtylene	POP	GC	6.14	positive	152.0 ➔ 151.0	25	152.0 ➔ 126.0	30	70	0.9673	143.84	0.6
6	Acephate	P	LC	1.65	positive	184.0 ➔ 143.0	15	143.0 ➔ 95.0	15	70	0.9941	109.14	2.5
7	Acetaminophen (Paracetemol)	M	LC	2.76	positive	152.1 ➔ 65.0	40	152.1 ➔ 93.0	20	150	0.9881	160.9	1.25
8	Acetamiprid	P	LC	4.44	positive	223.1 ➔ 126.0	27	223.1 ➔ 90.0	45	140	0.9981	95.84	0.3
9	Acrinathrin	P	LC	10.71	positive	559.0 ➔ 208.0	10	559.0 ➔ 181.0	30	70	0.9904	89.03	0.6
10	Albendazole	M	LC	7.26	positive	266.1 ➔ 234.1	16	266.1 ➔ 191.0	32	155	0.9966	90.75	0.15
11	Aldicarb	P	LC	5.17	positive	208.0 ➔ 116.0	10	116.0 ➔ 89.1	4	100	0.9979	84.14	0.15
12	Aldicarb-sulfone	P	LC	2.8	positive	240.1 ➔ 76.0	16	223.1 ➔ 86.1	13	75	0.9966	90.21	0.6
13	Aldicarb-sulfoxide	P	LC	2.75	positive	207.1 ➔ 131.9	10	207.1 ➔ 89.1	10	86	0.9972	92.14	1.25
14	Aldrin	POP	GC	9.89	positive	255.0 ➔ 220.0	25	263.0 ➔ 228.0	10	70	0.9840	116.27	0.3
15	Anthracene	POP	GC	8.4	positive	178.0 ➔ 176.0	35	178.0 ➔ 152.0	30	70	0.9868	133.33	0.6
16	Atrazine	P	LC	6.77	positive	216.0 ➔ 173.9	15	216.0 ➔ 103.8	30	130	0.9974	85.71	0.15
17	Azinphos-methyl	P	LC	7.28	positive	318.0 ➔ 132.1	8	340.0 ➔ 160.0	10	60	0.9969	100.46	0.15
18	Azoxystrobin	P	LC	7.59	positive	404.1 ➔ 372.1	8	404.1 ➔ 344.1	24	110	0.9985	89.93	0.15
19	BDE-28	POP	GC	12.23	positive	406.0 ➔ 246.0	20	406.0 ➔ 167.0	25	70	0.9768	124.38	0.15
20	BDE-47	POP	GC	14.32	positive	326.0 ➔ 138.0	45	484.0 ➔324.0	25	70	0.9824	128.48	0.3
21	BDE-85	POP	GC	17.1	positive	564.0 ➔ 404.0	25	566.0 ➔ 406.0	25	70	0.9618	128.8	0.3
22	BDE-99	POP	GC	16.28	positive	566.0 ➔ 406.0	25	564.0 ➔ 404.0	30	70	0.9758	141.83	0.3
23	BDE-100	POP	GC	15.86	positive	566.0 ➔ 406.0	25	564.0 ➔ 404.0	25	70	0.9698	147.25	0.3
24	BDE-153	POP	GC	18.06	positive	644.0 ➔ 484.0	25	486.0 ➔ 377.0	30	70	0.9792	141.23	0.15
25	BDE-154	POP	GC	17.5	positive	644.0 ➔ 484.0	25	486.0 ➔ 377.0	30	70	0.9776	149.11	0.3
26	BDE-183	POP	GC	20.14	positive	561.6 ➔ 454.7	40	563.6 ➔ 454.7	40	70	0.9649	233.14	0.15
27	Benalaxyl	P	LC	8.98	positive	326.2 ➔ 148.0	20	326.2 ➔ 208.0	12	90	0.9985	82.41	0.15
28	Bendiocarb	P	LC	5.92	positive	224.1 ➔ 166.9	8	224.2 ➔ 108.9	15	120	0.9977	101.28	0.3
29	Bendiocarb metabolite (2, 2-dimethylbenzo-1, 3-dioxol-4-ol)	P	GC	4.83	positive	166.0 ➔ 151.0	10	166.0 ➔ 126.0	20	70	0.9559	347.74	2.5
30	Benfuracarb	P	LC	9.73	positive	411.2 ➔ 190.0	13	411.2 ➔ 252.0	15	110	0.9304	22.53	1.25
31	Benzo[a]anthracene	POP	GC	13.88	positive	228.0 ➔ 226.0	40	228.0 ➔ 202.0	35	70	0.9857	126.76	0.15
32	Benzo[a]pyrene	POP	GC	16.91	positive	252.0 ➔ 250.0	45	252.0 ➔ 248.0	60	70	0.9863	138.96	0.15
33	Benzo[b]fluoranthene	POP	GC	16.27	positive	252.0 ➔ 248.0	60	252.0 ➔ 226.0	35	70	0.9796	141.04	0.3
34	Benzo[ghi]perylene	POP	GC	19.65	positive	276.0 ➔ 274.0	50	276.0 ➔ 272.0	60	70	0.9828	129.94	0.15
35	Benzo[k]fluoranthene	POP	GC	16.3	positive	252.0 ➔ 250.0	45	252.0 ➔ 224.0	40	70	0.9801	125.79	0.3
36	Bifenthrin	P	GC	13.89	positive	440.0 ➔ 181.0	5	440.0 ➔ 165.0	60	94	0.9893	99.72	0.15
37	Bitertanol	P	LC	9.22	positive	338.2 ➔ 70.0	4	338.2 ➔ 269.2	5	100	0.9961	102.64	0.3
38	Boscalid (formerly nicobifen)	P	GC	16.55	positive	3434.0 ➔ 272.0	30	343.0 ➔ 140.0	45	100	0.9721	100.39	0.15
39	Brodifacoum	AR	LC	10.64	negative	521.3 ➔ 79.0	50	523.3 ➔ 135.0	45	220	0.9879	36.3	0.3
40	Bromadiolone	AR	LC	9.7	negative	525.3 ➔ 250.0	40	527.3 ➔ 250.0	40	200	0.9892	88	0.3
41	Bromopropylate	P	GC	13.87	positive	341.0 ➔ 183.0	15	341.0 ➔ 157.0	45	70	0.9760	100.52	0.15
42	Bromuconazole (two isomers)	P	GC	13.81	positive	295.0 ➔ 173.0	10	295.0 ➔ 175.0	10	70	0.9764	97.2	0.3
43	Bupirimate	P	LC	8.4	positive	273.0 ➔ 108.0	15	273.0 ➔ 193.0	5	70	0.9953	96.56	0.3
44	Buprofezin	P	LC	9.88	positive	306.1 ➔ 201.0	12	306.1 ➔ 116.0	12	140	0.9975	79.09	0.15
45	Cadusafos (ebufos)	P	LC	9.4	positive	271.1 ➔ 159.0	16	271.1 ➔ 131.0	22	100	0.9903	88.95	0.3
46	Carbaryl	P	LC	6.24	positive	202.1 ➔ 145.1	4	202.1 ➔ 127.1	28	95	0.9968	101.8	0.15
47	Carbendazim (azole)	P	LC	3.4	positive	192.1 ➔ 160.1	4	202.1 ➔ 127.1	28	90	0.9977	70.73	0.3
48	Carbofuran	P	LC	5.95	positive	222.1 ➔ 123.1	20	222.1 ➔ 165.1	30	80	0.9973	93.77	0.15
49	Carbofuran-3-hydroxy	P	LC	4.27	positive	238.1 ➔ 163.1	10	238.1 ➔ 181.1	10	110	0.9976	79.76	0.6
50	Cefuroxima axetil (two isomers)	M	LC	5.4	positive	533.0 ➔ 447.0	15	533.0 ➔ 386.0	20	160	0.9970	197.75	0.3
51	Chloramphenicol	M	LC	4.62	negative	321.0 ➔ 152.1	4	323.0 ➔ 152.1	4	113	0.9766	115.61	2.5
52	Chlorantraniliprole	P	LC	7.33	positive	483.9 ➔ 452.9	16	483.9 ➔ 285.9	8	105	0.9979	101.23	0.3
53	Chlorfenapyr	P	GC	12.01	positive	247.0 ➔ 200.0	30	247.0 ➔ 227.0	15	70	0.9864	93.61	0.6
54	Chlorfenvinphos	P	LC	9.08	positive	361.1 ➔ 98.9	34	358.9 ➔ 155.1	8	105	0.9947	96.77	0.3
55	Chlorobenzilate	P	GC	12.14	positive	251.0 ➔ 111.0	40	251.0 ➔ 139.0	15	70	0.9866	93.38	0.15
56	Chlorophacinone	AR	LC	8.75	negative	373.2 ➔ 201.0	20	375.2 ➔ 203.0	20	160	0.9345	72.97	5
57	Chlorpropham	P	GC	7.12	positive	213.0 ➔ 127.0	15	153.0 ➔ 90.0	25	70	0.9818	109.49	0.15
58	Chlorpyrifos	P	GC	9.93	positive	314.0 ➔ 258.0	15	314.0 ➔ 286.0	5	70	0.9798	98.63	0.15
59	Chlorpyrifos methyl	P	GC	9.12	positive	286.0 ➔ 93.0	25	286.0 ➔ 271.0	15	70	0.9839	87.88	0.15
60	Chlorthal dimethyl	POP	GC	10.03	positive	300.9 ➔ 166.9	55	300.9 ➔ 222.9	25	70	0.9757	103.06	0.15
61	Chrysene	M	GC	13.95	positive	228.0 ➔ 226.0	40	228.0 ➔ 227.0	25	70	0.9800	123.04	0.3
62	Clindamycin	P	LC	5.65	positive	425.2 ➔ 126.1	20	425.2 ➔ 377.2	20	150	0.9969	98.59	1.25
63	Clofentezine	P	LC	9.2	positive	303.1 ➔ 138.0	12	303.1 ➔ 102.0	40	120	0.9940	92.23	0.3
64	Clothianidin	M	LC	3.9	positive	250.0 ➔ 169.0	8	250.0 ➔ 131.9	8	100	0.9959	109.87	1.25
65	Cortiscosterone 21 acetate	P	LC	7.89	positive	389.1 ➔ 329.0	13	389.1 ➔ 371.0	13	80	0.9968	89.53	1.25
66	Coumachlor	P	LC	8.6	positive	343.1 ➔ 162.8	15	342.1 ➔ 285.0	15	120	0.9849	94.86	0.6
67	Coumaphos	AR	LC	8.99	positive	363.0 ➔ 227.0	30	363.0 ➔ 306.9	15	120	0.9912	107.11	0.6
68	Coumatetralyl	P	LC	8.26	negative	291.1 ➔ 141.0	30	291.1 ➔ 247.0	20	140	0.9922	93.55	0.6
69	Cyazofamid	P	LC	8.48	positive	325.0 ➔ 108.0	20	325.0 ➔ 261.1	15	90	0.9946	90.54	0.6
70	Cyflufenamid	P	LC	9.19	positive	413.1 ➔ 223.1	33	413.1 ➔ 295.1	23	70	0.9952	89.99	0.3
71	Cyfluthrin (sum of four isomers)	P	GC	16.21	positive	226.0 ➔ 206.0	25	198.9 ➔ 170.1	25	70	0.9735	76.97	2.5
72	Cyhalothrin (lambda isomer)	P	LC	10.48	positive	181.1 ➔ 152.1	10	181.1 -> 127.1	46	70	0.9883	97.58	2.5
73	Cymoxanil	P	LC	4.7	positive	199.1 ➔ 128.0	4	199.1 ➔ 110.9	12	90	0.9976	92.87	1.25
74	Cypermethrin (sum of four isomers)	P	GC	16.54	positive	163.0 ➔ 109.0	20	163.0 ➔ 127.0	5	70	0.9786	66.91	1.25
75	Cyproconazole (two isomers)	P	LC	8.14	positive	292.2 ➔ 70.2	18	292.2 ➔ 125.1	24	100	0.9971	128.58	0.3
76	Cyprodinil	P	LC	8.57	positive	226.0 ➔ 93.0	33	226.0 ➔ 108	25	100	0.9914	60.14	0.6
77	Cyromazine	M	LC	1.23	positive	167.1 ➔ 85.0	16	167.1 ➔ 125.0	20	120	0.9942	33.21	2.5
78	Danofloxacin	P	LC	3.53	positive	358.2 ➔ 340.1	20	358.2 ➔ 82.1	50	159	0.9495	210.36	2.5
79	Dazomet	POP	GC	7.81	positive	161.9 ➔ 44.0	28	161.9 ➔ 89.0	5	70	0.9830	116.07	0.3
80	Deltamethrin	POP	LC	10.64	positive	523.0 ➔ 281.0	10	523.0 ➔ 506.0	5	100	0.9775	79.77	1.25
81	Demeton-S-methyl	POP	LC	6	positive	230.9 ➔ 88.9	5	230.9 ➔ 61.0	30	50	0.9968	88.14	0.15
82	Demeton-S-methyl-sulfone (Dioxydemeton)	P	LC	3.3	positive	263.0 ➔ 169.0	24	263.0 ➔ 109.0	12	120	0.9974	91.79	0.6
83	Dexamethasone	P	LC	7.16	positive	393.2 ➔ 373.2	2	393.2 ➔ 355.2	6	103	0.9924	102.52	1.25
84	Diazinon	P	GC	8.28	positive	137.1 ➔ 54.0	20	304.0 ➔ 179.0	15	70	0.9710	102.06	0.15
85	Dibenzo[a,h]anthracene	M	GC	19.18	positive	278.0 ➔ 276.0	40	278.0 ➔ 250.0	60	70	0.9866	131.16	0.15
86	Dichlorodiphenyldichloroethane (p,p′ DDD)	P	GC	12.32	positive	235.0 ➔ 165.0	20	235.0 ➔ 199.0	15	70	0.9898	127.87	0.15
87	Dichlorodiphenyldichloroethylene (p,p′ DDE)	POP	GC	11.58	positive	318.0 ➔ 176.0	60	318.0 ➔ 248.0	30	70	0.9581	116.04	0.3
88	Dichlorodiphenyltrichloroethane (p,p′ DDT)	P	GC	12.98	positive	235.0 ➔ 165.0	40	235.0 ➔ 199.0	15	70	0.9435	14.81	1.25
89	Diclofenac	P	LC	8.73	positive	296.0 ➔ 215.1	16	296.0 ➔ 214.1	48	103	0.9839	71.47	2.5
90	Dicloran	M	GC	7.8	positive	206.0 ➔ 176.0	10	206.0 ➔ 148.0	25	70	0.9872	106.98	0.6
91	Dichlorvos	POP	LC	5.79	positive	221.0 ➔ 79.1	28	221.0 ➔ 109.1	16	105	0.9971	42.69	0.6
92	Dieldrin	P	GC	11.67	positive	263.0 ➔ 228.0	15	277.0 ➔ 241.0	15	70	0.9724	111.8	1.25
93	Diethathyl ethyl	P	LC	8.73	positive	312.2 ➔ 238.1	15	312.2 ➔ 162.0	30	120	0.9974	84.45	0.15
94	Diethofencarb	AR	LC	7.59	positive	268.2 ➔ 226.1	5	268.2 ➔ 152.0	20	110	0.9949	102.38	0.15
95	Difenacoum	P	LC	10.25	negative	443.2 ➔ 135.0	40	443.2 ➔ 293.0	35	200	0.9926	79.52	0.3
96	Difenoconazole	AR	LC	9.41	positive	406.1 ➔ 250.9	28	406.1 ➔ 337.0	16	176	0.9963	118.11	0.3
97	Difethialone	M	LC	10.8	negative	537.3 ➔ 79.0	50	537.3 ➔ 151.0	45	220	0.9892	38.64	0.6
98	Difloxacin	P	LC	3.85	positive	400.2 ➔ 382.1	20	400.2 ➔ 356.1	16	149	0.9692	234.88	2.5
99	Diflubenzuron	P	LC	8.63	positive	311.0 ➔ 158.0	8	311.0 ➔ 141.0	32	90	0.9742	85.04	1.25
100	Diflufenican	P	GC	13.27	positive	394.0 ➔ 266.0	10	266.0 -> 246.0	10	70	0.9810	97.76	0.15
101	Dimethenamid-P (and its R-isomer)	P	LC	7.72	positive	276.1 ➔ 244.1	10	276.1 ➔ 168.1	20	125	0.9979	92.69	0.15
102	Dimethoate	P	LC	4.2	positive	230.0 ➔ 125.0	16	230.0 ➔ 198.8	20	70	0.9976	63.84	0.6
103	Dimethomorph (two isomers)	P	LC	7.87	positive	388.1 ➔ 301.1	20	388.1 ➔ 165.1	32	180	0.9955	121.84	0.3
104	Dimethylphenylsulfamide (DMSA, metabolite of dichlofluanid)	P	LC	5.24	positive	201.1 ➔ 92.1	15	201.1 ➔ 137.1	5	100	0.9954	63.21	1.25
105	Diniconazole-M	P	GC	12.27	positive	326.1 ➔ 70.0	15	328.1 ➔ 70.0	15	70	0.9838	85.44	0.3
106	Dinocap	AR	LC	10.43	negative	295.4 ➔ 208.9	30	295.4 ➔ 193.0	35	150	0.9819	68.11	1.25
107	Diphacinone	P	LC	8.45	negative	339.1 ➔ 167.0	25	339.1 ➔ 145.0	20	170	0.9348	94.92	5
108	Diphenylamine	P	GC	6.97	positive	168.0 ➔ 167.2	15	169.0 ➔ 66.0	15	70	0.9805	116.63	0.3
109	N,N-dimethylformamidine (DMF, metabolite of amitraz)	P	LC	5.48	positive	150.1 ➔ 77.0	40	149.9 ➔ 105.8	30	100	0.9878	166.59	1.25
110	Dodine	P	LC	9.1	positive	228.3 ➔ 43.0	40	228.3 ➔ 57.0	25	150	0.9965	154.55	0.6
111	Endosulfan alfa	P	GC	11.21	positive	241.0 ➔ 206.0	15	195.0 ➔ 160.0	10	70	0.9789	97.34	0.3
112	Endosulfan beta	P	GC	12.22	positive	241.0 ➔ 206.0	15	195.0 ➔ 159.0	15	70	0.9836	101.02	0.3
113	Endosulfan sulfate	P	GC	12.97	positive	270.0 ➔ 235.0	15	387.0 ➔ 289.0	5	70	0.9251	38.28	0.3
114	Endrin	P	GC	12.05	positive	263.0 ➔ 193.0	35	245.0 ➔ 173.0	25	70	0.9603	114.03	1.25
115	Enrofloxacin	P	LC	3.62	positive	360.2 ➔ 316.1	16	360.2 ➔ 245.1	28	144	0.9308	139.22	2.5
116	EPN	POP	GC	13.9	positive	157.0 ➔ 63.0	10	157.0 ➔ 110.0	15	70	0.9677	75.05	0.3
117	Epoxiconazole	M	LC	8.47	positive	330.0 ➔ 120.9	24	330.1 ➔ 100.9	50	120	0.9961	91.32	0.3
118	Eprinomectin	P	LC	10.83	positive	878.5 ➔ 186.0	15	936.5 ➔ 490.4	60	160	0.9844	106.34	2.5
119	Eritromicin	P	LC	6.83	positive	734.5 ➔ 158.1	32	734.5 ➔ 576.3	16	172	0.9955	102.43	0.3
120	Esfenvalerate	M	GC	17.56	positive	167.1 ➔ 125.1	15	167.1 ➔ 89.1	45	70	0.9855	59.05	1.25
121	Ethion (diethion)	M	LC	10.02	positive	385.0 ➔ 199.0	5	385.0 ➔ 171.0	10	100	0.9944	92.79	0.15
122	Ethirimol	P	LC	4.8	positive	210.2 ➔ 140.1	20	210.2 ➔ 98.1	28	160	0.9972	79.49	0.6
123	Ethofumesate	P	GC	9.59	positive	286.0 ➔ 207.0	5	286.0 ➔ 161.0	20	70	0.9801	102.24	0.15
124	Ethoprophos	P	LC	8.41	positive	243.1 ➔ 97.0	30	243.1 ➔ 130.9	15	90	0.9954	96.81	0.3
125	Etofenprox	P	GC	16.75	positive	163.0 ➔ 107.0	20	163.0 ➔ 135.0	10	70	0.9858	102.63	0.3
126	Etoxazole	P	LC	10.33	positive	360.1 ➔ 141.0	26	360.1 ➔ 304.0	16	160	0.9974	80.95	0.15
127	Famoxadone	P	LC	9.05	positive	392.1 ➔ 330.9	5	392.2 ➔ 238.1	12	110	0.9915	89.82	0.6
128	Fenamidone	P	LC	7.73	positive	392.1 ➔ 330.9	5	392.1 ➔ 238.1	12	110	0.9883	105.31	0.3
129	Fenamiphos	P	LC	8.64	positive	304.1 ➔ 217.1	20	304.1 ➔ 202.0	36	120	0.9960	89.88	0.3
130	Fenamiphos sulfone	P	LC	6.17	positive	336.1 ➔ 188.0	31	336.1 ➔ 266.0	23	120	0.9964	126.5	0.3
131	Fenamiphos sulfoxide	P	LC	5.92	positive	320.1 ➔ 233.0	20	320.1 ➔ 108.1	44	120	0.9950	136.43	1.25
132	Fenarimol	P	GC	15.04	positive	139.0 ➔ 75.0	30	139.0 ➔ 111.0	15	70	0.9933	99.43	0.15
133	Fenazaquin	P	LC	10.75	positive	307.2 ➔ 57.1	25	307.2 ➔ 161.1	16	90	0.9776	43.22	0.15
134	Fenbendazole	P	LC	8.09	positive	300.1 ➔ 268.1	20	300.1 ➔ 159.0	36	156	0.9955	88.98	0.15
135	Fenbuconazole	P	GC	16.18	positive	198.0 ➔ 102.0	30	198.0 ➔ 78.0	30	70	0.9795	74.4	0.3
136	Fenbutatin oxide	M	LC	11.61	positive	519.0 ➔ 197.0	55	517.3 ➔ 194.9	60	180	0.9840	63.62	0.3
137	Fenhexamid	P	LC	8.35	positive	302.1 ➔ 97.1	20	302.1 ➔ 55.1	40	130	0.9892	86.52	1.25
138	Fenitrothion	P	GC	9.57	positive	277.0 ➔ 109.0	15	277.0 ➔ 125.0	15	70	0.9591	90.52	1.25
139	Fenoxycarb	P	LC	8.69	positive	302.1 ➔ 88.0	20	302.1 ➔ 116.1	10	110	0.9939	97.48	0.3
140	Fenpropathrin	P	LC	14	positive	367.2 ➔ 125.0	16	350.0 ➔ 125.0	16	72	0.9829	95.37	0.6
141	Fenpropidin	P	LC	7.25	positive	274.3 ➔ 147.0	30	274.3 ➔ 86.0	25	170	0.9968	102.13	0.15
142	Fenpropimorph	P	LC	7.51	positive	304.3 ➔ 147.1	30	304.3 ➔ 130.0	25	120	0.9970	98.38	0.15
143	Fenpyroximate	P	LC	10.49	positive	422.2 ➔ 366.2	12	422.2 ➔ 135.0	36	160	0.9951	86.38	0.3
144	Fenthion	P	GC	9.89	positive	278.0 ➔ 109.0	15	278.0 -> 125.0	20	70	0.9909	100.18	0.15
145	Fenthion oxon	P	LC	7.33	positive	263.1 ➔ 231.2	16	263.1 ➔ 216.0	24	120	0.9964	97.32	0.3
146	Fenthion oxon sulfone	P	LC	4.5	positive	295.0 ➔ 217.0	15	295.0 ➔ 104.2	24	110	0.9973	109.02	0.6
147	Fenthion oxon sulfoxide	P	LC	4.25	positive	279.0 ➔ 264.2	20	279.0 ➔ 104.1	28	110	0.9977	84.23	0.6
148	Fenthion sulfone	P	LC	6.39	positive	311.0 ➔ 125.0	22	311.0 ➔ 109.0	28	140	0.9943	115.8	1.25
149	Fenthion sulfoxide	P	LC	6.16	positive	295.0 ➔ 108.9	30	295.0 ➔ 280.0	18	140	0.9966	118.68	0.6
150	Fenvalerate	P	GC	17.37	positive	167.0 ➔ 125.1	22	167.0 ➔ 89.0	30	70	0.9852	68.95	1.25
151	Fipronil	P	LC	10.63	negative	435.0 ➔ 330.0	12	435.0 ➔ 249.9	26	116	0.9740	94.67	0.15
152	Fipronil sulfide	P	GC	10.55	positive	351.0 ➔ 255.0	20	420.0 ➔ 351.0	25	70	0.9687	136.6	1.25
153	Flocoumafen	P	LC	10.35	negative	541.3 ➔ 382.0	25	541.3 ➔ 161.0	40	230	0.9865	63.98	0.3
154	Fluazinam	P	LC	10	negative	462.9 ➔ 416.0	10	462.9 ➔ 398.0	9	140	0.9966	90.09	0.6
155	Flubendiamide	AR	LC	8.79	positive	408.0 ➔ 274.0	15	408.0 ➔ 256.0	30	120	0.9924	117.11	1.25
156	Flucythrinate (two isomers)	P	GC	16.68	positive	156.9 ➔ 107.1	15	199.1 ➔ 107.1	25	70	0.9866	79.25	0.15
157	Fludioxonil	P	GC	11.52	positive	248.0 ➔ 127.0	30	248.1 ➔ 182.1	10	70	0.9891	90.92	0.15
158	Flufenoxuron	P	LC	10.34	positive	489.1 ➔ 158.0	20	489.1 ➔ 140.9	56	110	0.9874	92.41	0.6
159	Flumequine	P	LC	6.12	positive	262.1 ➔ 244.0	16	262.1 ➔ 202.0	32	116	0.9947	134.56	0.3
160	Flunixin	P	LC	8.1	positive	297.1 ➔ 279.1	24	297.1 ➔ 264.1	32	141	0.9957	30.22	0.6
161	Fluopyram	M	GC	10.62	positive	173.0 ➔ 95.0	35	223.0 ➔ 196.0	40	70	0.9866	96.79	0.15
162	Fluoranthene	M	GC	10.68	positive	202.0 ➔ 201.0	27	202.0 ➔ 152.0	42	70	0.9837	118.33	0.15
163	Fluorene	P	GC	6.8	positive	165.0 ➔ 163.0	40	165.0 ➔ 139.0	30	70	0.9736	121.63	0.6
164	Fluquinconazole	POP	GC	15.81	positive	340.0 ➔ 298.0	15	340.0 ➔ 286.0	25	70	0.9608	107.7	0.3
165	Flusilazole	POP	LC	8.65	positive	316.1 ➔ 247.1	15	316.1 ➔ 165.0	20	160	0.9899	86.47	0.3
166	Flutolanil	P	LC	7.93	positive	324.1 ➔ 262.1	16	324.1 ➔ 242.1	24	130	0.9979	92.71	0.15
167	Flutriafol	P	GC	11.26	positive	219.0 ➔ 95.0	35	219.0 ➔ 123.0	15	70	0.9910	95.35	0.3
168	Fluvalinate tau	P	GC	17.57	positive	250.1 ➔ 55.1	30	252.0 ➔ 200.0	20	70	0.9754	40.31	1.25
169	Fonofos	P	GC	8.24	positive	246.0 ➔ 109.0	15	246.0 ➔ 237.0	5	70	0.9814	105.24	0.3
170	Formetanate	P	LC	1.77	positive	222.1 ➔ 165.1	12	222.1 ➔ 46.2	28	105	0.9290	72.76	2.5
171	Fosthiazate	P	LC	6.53	positive	284.0 ➔ 104.0	20	284.0 ➔ 227.8	8	90	0.9975	94.08	0.15
172	Heptachlor	P	GC	9.3	positive	272.0 ➔ 237.0	15	274.0 ➔ 239.0	15	70	0.9806	78.64	0.15
173	Hexachlorobencene	P	GC	7.76	positive	284.0 ➔ 214.0	40	284.0 ➔ 249.0	25	70	0.9825	117.77	0.3
174	Hexachlorocyclohexane (alpha)	POP	GC	7.63	positive	219.0 ➔ 109.0	10	219.0 ➔ 183.0	10	70	0.9891	111.63	0.3
175	Hexachlorocyclohexane (beta)	POP	GC	8.03	positive	219.0 ➔ 109.0	40	219.0 ➔ 183.0	5	70	0.9772	92.19	0.3
176	Hexachlorocyclohexane (delta)	POP	GC	8.5	positive	219.0 ➔ 109.0	45	219.0 ➔ 183.0	5	70	0.9771	62.23	0.6
177	Hexaclorocyclohexane (gamma, lindane)	POP	GC	8.13	positive	291.0 ➔ 109.0	40	219.0 ➔183.0	10	70	0.9682	75.72	1.25
178	Hexaconazole (two isomers)	POP	LC	8.49	positive	314.1 ➔ 70.1	20	316.1 ➔ 70.1	20	95	0.9844	100.39	0.3
179	Hexaflumuron	P	LC	9.57	negative	458.8 ➔ 439.0	8	458.8 ➔ 175.0	30	100	0.9918	105.98	0.6
180	Hexythiazox	P	LC	10.18	positive	353.1 ➔ 227.9	8	353.1 ➔ 168.1	24	120	0.9946	92.42	0.15
181	Imazalil (enilconazole)	P	LC	6.53	positive	297.1 ➔ 159.0	20	297.1 ➔ 69.1	18	100	0.9972	97.09	0.15
182	Imidacloprid	P	LC	4.1	positive	256.0 ➔ 175.0	12	256.0 ➔ 209.0	12	110	0.9951	224	0.6
183	Indeno [1,2,3-cd] pyrene	P	GC	19.11	positive	276.0 ➔ 274.0	50	276.0 ➔ 272.0	60	70	0.9812	131.78	0.3
184	Indoxacarb	POP	LC	9.47	positive	528.1 ➔ 293.1	10	528.1 ➔ 202.8	48	140	0.9883	181.2	0.6
185	Iprodione	P	GC	13.67	positive	314.0 ➔ 56.0	20	314.0 ➔ 245.0	10	70	0.9588	29.79	1.25
186	Iprovalicarb	P	LC	8.2	positive	321.2 ➔ 119.0	15	321.2 ➔ 202.9	20	110	0.9913	101.06	0.3
187	Isocarbophos	P	GC	10.37	positive	230.0 ➔ 155.0	25	230.0 ➔ 198.0	10	70	0.9856	99.29	0.3
188	Isofenphos methyl	P	LC	8.82	positive	199.0 ➔ 121.0	10	241.0 ➔ 121.0	25	70	0.9974	84.43	0.15
189	Isoprothiolane	P	GC	11.45	positive	291.1 ➔ 189.0	30	291.1 ➔ 145.0	36	100	0.9928	99.83	0.3
190	Ivermectin B1a	P	LC	11.5	positive	897.5 ➔ 753.5	50	897.5 ➔ 329.3	60	160	0.9657	65.26	1.25
191	Josamycin	M	LC	7.48	positive	860.5 ➔ 173.9	40	860.5 ➔ 108.9	40	200	0.9964	86.07	0.3
192	Ketoprofen	M	LC	7.34	positive	255.1 ➔ 209.1	8	255.1 ➔ 77.1	48	123	0.9949	114.88	1.25
193	Kresoxim methyl	M	LC	8.8	positive	116.0 ➔ 89.0	15	206.0 ➔ 131.0	10	70	0.9906	80.83	0.6
194	Leptophos	P	GC	14.58	positive	171.1 ➔ 77.1	15	377.0 ➔ 362.0	20	70	0.9664	80.36	0.3
195	Levamisole	P	LC	3.19	positive	205.1 ➔ 178.1	20	205.1 ➔ 123.0	32	141	0.9959	105.79	0.6
196	Lincomycin	M	LC	3.57	positive	407.2 ➔ 126.1	24	407.2 ➔ 359.2	16	150	0.9882	117.63	1.25
197	Linuron	M	LC	7.56	positive	249.0 ➔ 160.1	20	249.0 ➔ 182.3	8	120	0.9963	93.64	0.6
198	Lufenuron	POP	LC	10.04	negative	509.0 ➔ 339.0	5	509.0 ➔ 326.1	15	90	0.9933	111.99	0.3
199	Malaoxon	P	LC	6.05	positive	315.1 ➔ 127.2	12	315.1 ➔ 99.1	36	120	0.9967	93.21	0.15
200	Malathion	P	LC	7.95	positive	348.0 ➔ 126.7	15	348.0 ➔ 285.0	8	100	0.9978	92.86	0.15
201	Mandipropamid	P	LC	7.9	positive	412.1 ➔ 328.1	8	412.1 ➔ 356.1	4	130	0.9950	101.16	0.15
202	Mebendazole	P	LC	6.7	positive	296.1 ➔ 264.1	20	296.1 ➔ 77.0	48	151	0.9978	96.88	0.3
203	Mefenamic acid	P	LC	9.5	positive	242.1 ➔ 209.1	28	242.1 ➔ 180.1	0	108	0.9857	120.65	0.6
204	Mefenoxam (metalaxyl-M)	M	LC	6.97	positive	280.0 ➔ 220.0	10	280.0 ➔ 192.0	15	110	0.9982	90.57	0.15
205	Meloxicam	M	LC	7.13	positive	352.5 ➔ 114.8	20	352.5 ➔ 140.8	20	130	0.9940	152.18	0.6
206	Mepanipyrim	P	GC	11.14	positive	222.0 ➔ 221.0	15	222.0 ➔ 207.0	15	70	0.9818	79.51	0.6
207	Mepiquat	M	LC	0.66	positive	114.0 ➔ 98.0	36	114.0 ➔ 70.0	45	100	0.9954	63.31	0.6
208	Metaflumizone	P	LC	9.92	negative	505.0 ➔ 302.0	14	541.0 ➔ 302.0	20	90	0.9974	108.71	0.3
209	Metalaxyl	P	GC	9.31	positive	234.0 ➔ 146.1	20	249.0 ➔ 146.0	20	70	0.9888	119.76	0.15
210	Metaldehyde	P	LC	3.94	positive	194.1 ➔ 61.9	5	194.1 ➔ 106.0	5	50	0.9973	74.2	1.25
211	Metconazole	P	LC	9.17	positive	320.1 ➔ 70.2	33	322.1 ➔ 70.2	24	250	0.9981	86.75	0.15
212	Methamidophos (two isomers)	P	LC	1.18	positive	142.0 ➔ 94.0	12	142.0 ➔ 125.0	12	85	0.9936	120.45	2.5
213	Methidathion	P	LC	7.13	positive	320.1 ➔ 144.8	8	320.1 ➔ 85.0	30	84	0.9982	87.88	0.15
214	Methiocarb	P	LC	7.68	positive	226.1 ➔ 169.0	4	226.1 ➔ 121.1	12	90	0.9960	108.04	0.3
215	Methiocarb-sufone	P	LC	4.52	positive	258.1 ➔ 201.1	8	258.1 ➔ 122.1	22	100	0.9967	152.19	1.25
216	Methiocarb-sulfoxide	P	LC	4.03	positive	242.0 ➔ 185.0	22	242.0 ➔ 122.0	28	90	0.9974	100.81	0.6
217	Methomyl	P	LC	3.22	positive	163.1 ➔ 88.0	5	163.0 ➔ 106.0	8	80	0.9977	67.78	0.6
218	Methoxyfenozide	P	LC	8	positive	369.2 ➔ 149.0	10	369.2 ➔ 313.1	15	85	0.9982	88.3	0.15
219	Metoxychlor	P	GC	13.98	positive	227.0 ➔ 141.0	20	227.0 ➔ 169.0	15	70	0.9648	55.26	1.25
220	Metrafenone	POP	LC	9.29	positive	409.1 ➔ 209.1	8	411.1 ➔ 209.1	12	108	0.9948	102.68	0.15
221	Metronidazole	P	LC	2.55	positive	172.1 ➔ 128.0	12	172.1 ➔ 82.1	24	98	0.9949	127.67	1.25
222	Mevinphos (phosdrin)	P	LC	4.43	positive	225.0 ➔ 193.1	15	225.0 ➔ 127.0	12	65	0.9961	79.85	1.25
223	Mirex	M	GC	14.8	positive	237.0 ➔ 143.0	30	274.0 ➔ 237.0	10	70	0.9774	84.26	0.3
224	Monocrotophos	P	LC	3.3	positive	224.1 ➔ 126.8	12	224.1 ➔ 98.1	15	100	0.9972	82.04	1.25
225	Moxidectin	POP	LC	11.26	positive	641.4 ➔ 529.2	5	641.4 ➔ 499.2	5	100	0.9753	106.29	1.25
226	Myclobutanil	P	LC	8.1	positive	289.1 ➔ 70.1	16	289.1 ➔ 125.1	32	110	0.9977	183.45	0.15
227	N-(2,4-dimethylphenyl)-N′-methylformamidine (DMPF, metabolite of amitraz)	M	LC	3.35	positive	163.1 ➔ 122.1	15	163.1 ➔ 107.1	15	100	0.9842	104.84	2.5
228	N,N-Dimethyl-N′-p-tolylsulphamide (DMST, metabolite of tolyfluanid)	P	LC	6.09	positive	215.1 ➔ 106.1	10	215.1 ➔ 151.1	4	90	0.9949	111.49	0.6
229	Naphtalene	POP	GC	4.45	positive	128.0 ➔ 127.0	15	128.0 ➔ 102.0	25	70	0.9717	160.38	1.25
230	Naproxen	M	LC	7.59	positive	231.0 ➔ 185.0	10	231.1 ➔ 169.9	21	120	0.9764	409.72	5
231	Novobiocin	M	LC	9.62	positive	613.2 ➔ 218.1	10	613.2 ➔ 396.1	10	150	0.9812	175.16	2.5
232	Nuarimol	P	GC	13.24	positive	235.0 ➔ 139.0	15	235.0 ➔ 111.0	40	70	0.9866	101.96	0.15
233	Ofurace	P	LC	12.75	positive	282.0 ➔ 159.9	20	282.0 ➔ 147.9	30	100	0.9720	57.23	0.3
234	Omethoate	P	LC	2.85	positive	214.1 ➔ 124.8	22	214.1 ➔ 183.0	5	100	0.9968	95.64	0.6
235	Oxadixyl	P	LC	5.46	positive	279.1 ➔ 219.2	5	279.1 ➔ 132.2	32	110	0.9973	88.73	0.6
236	Oxamyl	P	LC	2.87	positive	237.1 ➔ 72.0	12	237.1 ➔ 90.0	5	70	0.9984	88.54	0.3
237	Oxfendazole	M	LC	5.63	positive	316.1 ➔ 159.0	32	316.1 ➔ 191.1	16	166	0.9963	143.49	0.3
238	Oxolinic acid	P	LC	5.06	positive	262.1 ➔ 216.0	32	262.1 ➔ 160.0	36	110	0.9941	126.86	0.6
239	Oxydemeton methyl	M	LC	3	positive	247.0 ➔ 169.0	12	247.0 ➔ 109.0	24	100	0.9975	90.62	0.6
240	Oxyfluorfen	P	GC	11.69	positive	252.0 ➔ 146.0	40	300.0 ➔ 223.0	15	70	0.9829	76.28	0.3
241	Paclobutrazol	P	LC	11.05	positive	294.1 ➔ 70.1	16	294.1 ➔ 125.2	36	115	0.9850	79	0.15
242	Parathion methyl	P	GC	9.12	positive	263.0 ➔ 109.0	15	263.0 ➔ 79.0	30	70	0.9823	86.34	0.3
243	PCB 28	POP	GC	9.02	positive	256.0 ➔ 186.0	25	256.0 ➔ 151.0	50	70	0.9827	119.38	0.3
244	PCB 52	POP	GC	9.58	positive	292.0 ➔ 222.0	25	292.0 ➔ 220.0	25	70	0.9791	117.94	0.15
245	PCB 77	POP	GC	11.74	positive	292.0 ➔ 220.0	25	292.0 ➔ 222.0	25	70	0.9828	119	0.15
246	PCB 81	POP	GC	11.57	positive	292.0 ➔ 220.0	25	292.0 ➔ 222.0	25	70	0.9691	118.13	0.15
247	PCB 101	POP	GC	11.08	positive	326.0 ➔ 256.0	30	328.0 ➔ 256.0	30	70	0.9773	145.98	0.15
248	PCB 105	POP	GC	12.67	positive	326.0 ➔ 256.0	30	328.0 ➔ 256.0	30	70	0.9795	122.8	0.15
249	PCB 114	POP	GC	12.39	positive	326.0 ➔ 256.0	30	328.0 ➔ 256.0	30	70	0.9815	129.03	0.15
250	PCB 118	POP	GC	12.19	positive	326.0 ➔ 256.0	30	328.0 ➔ 256.0	30	70	0.9839	127.49	0.15
251	PCB 123	POP	GC	12.14	positive	326.0 ➔ 256.0	30	328.0 ➔ 256.0	30	70	0.9802	133.73	0.3
252	PCB 126	POP	GC	13.23	positive	326.0 ➔ 256.0	30	328.0 ➔ 256.0	30	70	0.9856	128.92	0.15
253	PCB 138	POP	GC	13.07	positive	360.0 ➔ 290.0	25	360.0 ➔ 288.0	25	70	0.9822	134.56	0.15
254	PCB 153	POP	GC	12.58	positive	360.0 ➔ 290.0	25	360.0 ➔ 288.0	25	70	0.9791	123.08	0.3
255	PCB 156	POP	GC	13.97	positive	360.0 ➔ 290.0	25	360.0 ➔ 288.0	25	70	0.9608	129.48	0.15
256	PCB 157	POP	GC	14.07	positive	360.0 ➔ 290.0	25	360.0 ➔ 288.0	25	70	0.9811	143.51	0.15
257	PCB 167	POP	GC	13.56	positive	360.0 ➔ 290.0	25	360.0 ➔ 288.0	25	70	0.9830	127.89	0.15
258	PCB 169	POP	GC	14.62	positive	360.0 ➔ 290.0	25	360.0 ➔ 288.0	25	70	0.9850	132.35	0.15
259	PCB 180	POP	GC	14.25	positive	394.0 ➔ 324.0	30	394.0 ➔ 322.0	30	70	0.9920	139.99	0.15
260	PCB 189	POP	GC	15.26	positive	394.0 ➔ 324.0	30	394.0 ➔ 322.0	30	70	0.9852	132.07	0.15
261	Penconazole	P	GC	10.51	positive	248.0 ➔ 157.0	30	248.0 ➔ 192.0	15	70	0.9881	101.88	0.15
262	Pencycuron	P	LC	9.32	positive	329.1 ➔ 125.1	24	329.1 ➔ 217.9	12	160	0.9975	81.56	0.15
263	Pendimethalin	P	GC	10.2	positive	252.0 ➔ 162.0	10	252.0 ➔ 191.0	5	70	0.9960	99.46	0.3
264	Penicilina V	M	LC	6.48	positive	383.2 ➔ 159.9	10	383.2 ➔ 113.9	40	130	0.9848	161.32	2.5
265	Permethrin	P	GC	15.7	positive	183.0 ➔ 128.0	15	183.1 ➔ 153.1	15	70	0.9796	95.57	1.25
266	Phenanthrene	POP	GC	8.33	positive	178.0 ➔ 176.0	35	178.0 ➔ 152.0	28	70	0.9854	140.09	0.3
267	Phenylbutazone	M	LC	8.24	positive	309.2 ➔ 160.2	20	309.2 ➔ 77.1	55	140	0.9492	43.54	2.5
268	Phosalone	P	LC	9.2	positive	385.1 ➔ 182.0	20	385.1 ➔ 110.9	55	80	0.9972	90.45	0.3
269	Phosmet	P	LC	7.35	positive	318.0 ➔ 159.9	16	318.0 ➔ 133.0	40	90	0.9963	98.52	0.6
270	Phosmet-oxon	P	LC	5.39	positive	302.0 ➔ 160.0	10	302.0 ➔ 133.0	38	60	0.9967	86.6	1.25
271	Pirimicarb	P	LC	5.31	positive	239.1 ➔ 72.1	20	239.1 ➔ 182.1	12	100	0.9968	88.23	0.15
272	Pirimicarb-desmethyl	P	LC	3.6	positive	225.1 ➔ 168.1	8	225.1 ➔ 72.1	20	100	0.9982	73.62	0.3
273	Pirimiphos ethyl	P	GC	10.26	positive	318.0 ➔ 166.0	15	318.0 ➔ 182.0	15	70	0.9794	95.24	0.15
274	Pirimiphos methyl	P	GC	9.57	positive	306.1 ➔ 164.0	20	306.1 ➔ 108.1	32	100	0.9881	95.62	0.15
275	Prochloraz	P	LC	9.11	positive	376.0 ➔ 308.0	10	376.0 ➔ 70.1	20	100	0.9949	96.56	0.15
276	Procymidone	P	GC	10.8	positive	283.0 ➔ 67.0	40	283.0 ➔ 68.0	25	70	0.9871	102.97	0.6
277	Profenofos	P	LC	9.75	positive	375.0 ➔ 305.0	20	373.0 ➔ 303.0	20	100	0.9948	84.29	0.3
278	Propamocarb	P	LC	2.93	positive	189.2 ➔ 102.0	12	189.2 ➔ 144.0	8	110	0.9969	124.45	0.6
279	Propargite	P	LC	10.35	positive	368.2 ➔ 231.1	4	368.2 ➔ 175.0	12	88	0.9973	79.42	0.15
280	Propiconazole	P	LC	9.03	positive	342.0 ➔ 69.0	21	342.0 ➔ 159.0	39	90	0.9953	90.91	0.6
281	Propoxur	P	LC	5.88	positive	210.1 ➔ 168.1	35	210.1 ➔ 65.1	40	70	0.9980	84.74	0.3
282	Propyzamide (pronamide)	P	LC	7.94	positive	256.1 ➔ 190.0	16	256.1 ➔ 173.0	25	90	0.9971	92.15	0.3
283	Proquinazid	P	LC	10.59	positive	288.0 ➔ 245.0	15	288.0 ➔ 217.0	30	70	0.9942	80.72	0.15
284	Prothioconazol	P	GC	11.86	positive	186.0 ➔ 49.0	20	186.0 ➔ 53.0	25	70	0.9923	96.14	0.15
285	Prothiophos	P	GC	11.46	positive	266.9 ➔ 221.0	35	162.0 ➔ 63.1	30	70	0.9875	95.4	0.3
286	Pymetrozine	P	LC	2.8	positive	218.1 ➔ 105.0	20	218.1 ➔ 78.0	52	120	0.9960	148.99	1.25
287	Pyraclostrobin	P	LC	9.15	positive	388.1 ➔ 193.8	8	388.1 ➔ 163.1	28	120	0.9980	84.91	0.15
288	Pyrazophos	P	LC	9.23	positive	374.1 ➔ 222.1	23	374.1 ➔ 194.0	32	100	0.9971	106.05	0.3
289	Pyrene	POP	GC	11.14	positive	202.0 ➔ 201.0	27	202.0 ➔ 200.0	45	70	0.9852	111.07	0.3
290	Pyridaben	P	LC	10.75	positive	365.2 ➔ 309.0	8	309.1 ➔ 147.0	16	168	0.9961	54.03	0.15
291	Pyridaphenthion	P	LC	8.11	positive	341.0 ➔ 189.0	22	341.0 ➔ 205.0	34	100	0.9980	101.11	0.3
292	Pyrimethanil	P	GC	8.27	positive	198.0 ➔ 118.0	40	198.0 ➔ 158.0	20	70	0.9876	107.48	0.15
293	Pyriproxifen	P	LC	10.08	positive	322.2 ➔ 96.0	12	322.2 ➔ 184.9	24	80	0.9981	82.74	0.15
294	Quinalfos	P	LC	8.75	positive	299.1 ➔ 96.9	30	299.1 ➔147.1	20	130	0.9953	91.26	0.3
295	Quinoxyfen	P	LC	10.13	positive	308.0 ➔ 197.0	32	308.2 ➔ 161.8	55	120	0.9954	81.86	0.3
296	Rifampicin	M	LC	7.99	positive	823.5 ➔ 791.4	15	823.5 ➔ 399.1	25	160	0.9758	75.59	2.5
297	Rotenone	P	LC	8.63	positive	395.1 ➔ 213.1	20	395.1 ➔ 192.1	25	150	0.9757	115.42	0.6
298	Roxithromycin	M	LC	7.75	positive	838.5 ➔ 158.1	40	838.5 ➔ 116.1	55	200	0.9940	113.62	0.6
299	Simazine	P	LC	5.87	positive	202.4 ➔ 68.1	30	202.4 ➔ 68.1	20	120	0.9982	70.91	0.3
300	Spinosad (two isomers)	P	LC	9.19	positive	732.4 ➔ 142.0	22	732.4 ➔ 98.0	60	130	0.9924	89.19	1.25
301	Spiramycin (two isomers)	M	LC	4.68	positive	439.1 ➔ 101.1	20	439.1 ➔ 88.0	50	70	0.9970	91.74	2.5
302	Spirodiclofen	P	LC	10.51	positive	411.1 ➔ 71.2	15	411.1 ➔ 313.0	5	110	0.9923	118.42	2.5
303	Spiromesifen	P	LC	10.28	positive	388.0 ➔ 273.0	25	273.0 ➔ 187.0	15	110	0.9956	75.77	0.6
304	Spiroxamine	P	LC	7.7	positive	298.3 ➔ 144.1	16	298.3 ➔100.1	32	120	0.9961	105.38	0.3
305	Strychnine	AR	LC	3.15	positive	335.1 ➔ 184.0	45	335.1 ➔ 156.0	40	105	0.9966	91.72	0.6
306	Sulfacetamide	M	LC	2.12	positive	215.3 ➔ 155.9	10	215.3 ➔ 92.0	20	90	0.9877	143.43	2.5
307	Sulfachloropiridacine	M	LC	3.75	positive	285.0 ➔ 156.0	12	285.0 ➔ 92.1	28	101	0.9979	285.3	0.6
308	Sulfadiacine	M	LC	2.86	positive	251.0 ➔ 92.0	28	251.0 ➔ 156.0	12	111	0.9918	229.63	1.25
309	Sulfadimetoxine	M	LC	4.81	positive	311.0 ➔ 92.0	32	311.0 ➔ 156.0	16	139	0.9976	229.39	0.3
310	Sulfadoxine	M	LC	4.15	positive	311.1 ➔ 92.0	32	311.1 ➔ 156.0	16	126	0.9977	167.2	0.3
311	Sulfameracine	M	LC	3.32	positive	265.0 ➔ 92.0	28	265.0 ➔ 156.0	12	126	0.9972	280.38	0.6
312	Sulfametacine	M	LC	3.7	positive	279.1 ➔ 186.0	12	279.1 ➔ 92.0	32	134	0.9970	293.67	0.6
313	Sulfametizole	M	LC	3.37	positive	271.0 ➔ 92.0	28	271.0 ➔ 155.9	8	103	0.9972	321.37	1.25
314	Sulfametoxazole	M	LC	3.96	positive	254.0 ➔ 92.0	28	254.0 ➔ 156.0	12	111	0.9968	235.15	0.6
315	Sulfametoxipiridacine	M	LC	3.77	positive	281.0 ➔ 155.9	12	281.0 ➔ 92.1	28	121	0.9963	257.12	0.6
316	Sulfamonomethoxine	M	LC	4.03	positive	281.1 ➔ 156.0	14	281.1 ➔ 92.1	32	120	0.9886	298.13	1.25
317	Sulfapyridine	M	LC	2.8	positive	250.0 ➔ 156.0	12	250.0 ➔ 92.0	28	126	0.9953	236.91	1.25
318	Sulfaquinoxaline	M	LC	5	positive	301.0 ➔ 156.0	12	301.0 ➔ 92.1	32	159	0.9945	257.62	0.6
319	Sulfatiazole	M	LC	3.05	positive	256.0 ➔ 92.0	28	256.0 ➔ 156.0	12	106	0.9924	395.5	1.25
320	Sulfisoxazole	M	LC	4.14	positive	268.0 ➔ 156.0	8	268.0 ➔ 92.1	24	106	0.9973	251.38	0.6
321	Tebuconazole	P	LC	8.92	positive	308.2 ➔ 70.2	22	308.2 ➔ 125.1	53	120	0.9978	70.16	0.6
322	Tebufenocide	P	LC	8.67	positive	353.1 ➔ 132.9	22	353.1 ➔ 297.1	20	90	0.9971	80.95	0.15
323	Tebufenpyrad	P	LC	14.07	positive	334.2 ➔ 117.0	47	334.2 ➔ 145.0	37	180	0.9846	104.68	0.15
324	Teflubenzuron	P	LC	10	negative	379.0 ➔ 339.0	15	379.0 ➔ 196.0	25	100	0.9912	77.79	1.25
325	Tefluthrin	P	GC	8.42	positive	177.0 ➔ 127.0	15	177.0 ➔ 87.0	15	70	0.9910	114.07	0.15
326	Telodrin (isobenzan)	P	GC	10.15	positive	310.8 ➔ 240.8	25	310.8 ➔ 274.8	5	70	0.9805	95.38	0.3
327	Terbufos	P	GC	8.15	positive	231.0 ➔ 97.0	20	231.0 ➔ 129.0	15	70	0.9904	102.34	0.15
328	Terbuthylazine	P	GC	7.74	positive	214.0 ➔ 104.0	20	214.0 ➔ 132.0	10	70	0.9975	81.85	0.15
329	Tetrachlorvinphos	P	LC	8.74	positive	367.0 ➔ 127.0	16	365.0 ➔ 127.0	16	110	0.9940	92.08	0.3
330	Tetraconazole	P	GC	10.03	positive	336.0 ➔ 204.0	35	336.0 ➔ 218.0	20	70	0.9793	102.89	0.3
331	Tetradifon	P	GC	14.36	positive	158.9 ➔ 111.0	20	354.0 ➔ 159.0	10	70	0.9910	104.79	0.15
332	Tetramethrin	P	GC	13.81	positive	164.0 ➔ 77.0	30	164.0 ➔ 107.0	15	70	0.9878	153.48	0.6
333	Thiabendazole	P	LC	3.9	positive	202.0 ➔ 175.0	24	202.0 ➔ 131.0	26	170	0.9980	71.17	0.3
334	Thiacloprid	P	LC	4.81	positive	253.0 ➔ 126.0	16	253.0 ➔ 90.0	40	140	0.9983	102.02	0.15
335	Thiamethoxam	P	LC	3.35	positive	292.0 ➔ 211.1	8	292.0 ➔ 132.0	22	80	0.9972	124.12	2.5
336	Thiophanate methyl	P	LC	5.89	positive	343.0 ➔ 151.0	20	343.0 ➔ 93.0	46	90	0.9976	191.68	0.3
337	Tolclofos methyl	M	GC	9.2	positive	265.0 ➔ 93.0	30	265.0 ➔ 220.0	25	70	0.9846	102.61	0.3
338	Tolfenamic acid	P	LC	9.77	negative	260.0 ➔ 216.1	8	260.0 ➔ 35.1	20	108	0.9872	543.45	1.25
339	Triadimefon	M	LC	8.05	positive	294.1 ➔ 69.3	20	294.1 ➔ 197.2	15	100	0.9948	197.11	0.3
340	Triadimenol	P	LC	8.23	positive	296.1 ➔ 70.0	10	298.1 ➔ 70.0	10	80	0.9968	130.33	0.6
341	Triazophos (hostathion)	P	LC	8.18	positive	314.1 ➔ 162.0	19	314.1 ➔ 118.9	35	100	0.9983	85.32	0.3
342	Trichlorfon	P	LC	4.05	positive	256.9 ➔ 109.0	12	258.9 ➔ 109.0	12	170	0.9966	191.7	1.25
343	Trifloxystrobin	P	LC	9.5	positive	409.1 ➔ 186.0	12	409.1 ➔ 145.0	52	110	0.9979	75.41	0.15
344	Triflumizole	P	LC	9.55	positive	346.1 ➔ 278.0	4	345.9 ➔ 73.0	15	80	0.9982	85.3	0.15
345	Triflumuron	P	LC	9.19	positive	359.0 ➔ 156.0	8	359.0 ➔ 139.0	32	120	0.9933	94.81	0.6
346	Trifluralin	P	GC	7.26	positive	264.0 ➔ 160.0	15	306.0 ➔ 264.0	5	70	0.9843	105.19	0.6
347	Trimethoprim	P	LC	3.08	positive	291.2 ➔ 123.0	24	291.2 ➔ 230.1	20	162	0.9959	85.63	0.6
348	Triticonazole	M	LC	8.4	positive	318.1 ➔ 70.1	33	320.1 ➔ 70.1	16	110	0.9760	119.71	1.25
349	Tylmicosin	P	LC	5.52	positive	869.6 ➔ 174.1	48	869.6 ➔ 696.4	44	294	0.9894	170.35	2.5
350	Tylosin	M	LC	6.85	positive	916.5 ➔ 174.1	40	916.5 ➔ 772.4	28	210	0.9966	92.36	1.25
351	Vinclozolin	P	GC	9.09	positive	212.0 ➔ 145.0	25	212.0 ➔ 109.0	50	70	0.9868	105.41	0.15
352	Warfarin	AR	LC	7.86	negative	307.1 ➔ 161.1	20	307.1 ➔ 250.1	20	140	0.9949	96.71	0.3
353	Zoxamide	P	LC	9.03	positive	336.0 ➔ 187.1	25	187.1 ➔ 88.9	40	200	0.9911	99.62	0.3

P (pesticide); M (medicament); POP (persistent organic pollutant); AR (anticoagulant rodenticide); LC (liquid chromatography); GC (gas chromatography).

## Data Availability

The data presented in this study are available on request from the corresponding author.

## Appendix A. Summary of Method Validation Results (Measured Uncertainty (MU), Recoveries and Inter- and Intraday Precisions (*n* = 5))

**No.**	**Compound**	**MU**	**0.3 ng/mL**			**1.25 ng/mL**			**5 ng/mL**			**20 ng/mL**			**40 ng/mL**		
				**Precision (RSD %)**			**Precision (RSD %)**			**Precision (RSD %)**			**Precision (RSD %)**			**Precision (RSD %)**	
			**Rec. (%)**	**Intraday**	**Interday**	**Rec. (%)**	**Intraday**	**Interday**	**Rec. (%)**	**Intraday**	**Interday**	**Rec. (%)**	**Intraday**	**Interday**	**Rec. (%)**	**Intraday**	**Interday**
1	2-Phenylphenol	22.71				111.20	11.92	11.29	93.26	17.78	20.06	100.24	17.65	18.54	98.44	7.68	8.21
2	4,4′-Dichlorobenzophenone (metabolite of dicofol)	24.86	93.72	17.18	19.30	113.48	15.09	14.00	89.56	13.23	15.55	99.40	14.69	15.56	103.47	8.37	8.51
3	Abamectine	31.79							97.94	13.71	14.74	98.51	11.04	11.80	101.43	10.98	11.40
4	Acenaphthene	25.01	94.41	15.26	17.02	97.82	4.08	4.40	94.69	11.06	12.29	96.61	18.23	19.86	101.84	8.60	8.89
5	Acenaphtylene	33.02				114.97	21.82	19.98	100.11	14.39	15.13	98.15	18.65	20.01	98.35	11.22	12.00
6	Acephate	16.55							97.41	9.85	10.65	98.76	3.85	4.10	101.74	5.62	5.82
7	Acetaminophen (Paracetemol)	16.63				118.40	15.13	13.45	87.58	11.04	13.27	99.23	7.33	7.77	102.05	5.61	5.78
8	Acetamiprid	7.32	107.58	10.19	9.97	98.11	2.90	3.11	94.21	6.35	7.09	102.36	4.51	4.64	99.41	2.48	2.63
9	Acrinathrin	25.44				87.18	12.90	15.57	96.81	13.01	14.15	100.45	8.45	8.86	98.88	8.67	9.23
10	Albendazole	10.42	93.43	7.73	8.70	104.12	3.76	3.81	102.98	4.43	4.52	103.28	5.39	5.50	96.07	2.30	2.52
11	Aldicarb	9.06	99.93	8.50	8.95	98.97	1.74	1.85	98.05	6.31	6.77	102.21	4.94	5.09	98.22	2.84	3.05
12	Aldicarb-sulfone	8.85				105.03	3.63	3.64	90.04	3.36	3.92	98.99	6.72	7.14	102.24	2.66	2.74
13	Aldicarb-sulfoxide	6.33				116.10	6.35	5.75	90.44	5.73	6.66	99.84	3.55	3.75	101.22	2.02	2.10
14	Aldrin	31.42	106.18	18.16	18.01	106.69	16.86	16.64	88.98	15.53	18.37	95.66	8.75	9.63	102.90	10.79	11.04
15	Anthracene	18.57				100.98	10.68	11.14	96.33	11.81	12.91	92.18	9.84	11.24	103.89	5.92	6.00
16	Atrazine	10.31	96.11	3.58	3.92	105.40	2.88	2.88	101.07	6.81	7.10	102.47	4.20	4.32	97.19	2.93	3.17
17	Azinphos-methyl	8.39	101.15	8.53	8.88	103.85	6.77	6.86	99.07	10.10	10.73	102.81	4.28	4.38	97.24	2.15	2.32
18	Azoxystrobin	10.50	98.04	6.27	6.73	104.37	2.98	3.01	97.56	6.32	6.82	99.06	5.25	5.58	100.42	3.61	3.79
19	BDE-28	36.70	104.31	14.98	15.12	101.58	7.56	7.83	90.05	16.18	18.91	93.67	13.52	15.19	105.17	12.45	12.46
20	BDE-47	32.46	99.47	11.23	11.88	111.52	2.64	2.49	96.16	15.98	17.49	97.65	14.95	16.12	100.18	11.19	11.75
21	BDE-85	58.35	94.45	19.12	21.30	104.21	17.67	17.85	92.60	10.45	11.88	94.08	10.62	11.89	100.70	20.16	21.08
22	BDE-99	42.81	104.66	20.57	20.68	103.83	13.88	14.08	90.67	12.91	14.99	90.83	10.23	11.86	105.41	14.66	14.63
23	BDE-100	52.30	96.85	12.38	13.46	107.88	19.14	18.67	96.30	17.82	19.48	97.14	10.98	11.90	99.85	18.00	18.97
24	BDE-153	36.24	102.12	18.29	18.85	104.09	16.37	16.56	95.49	18.09	19.94	99.65	14.59	15.41	101.80	12.53	12.96
25	BDE-154	43.70	97.71	13.27	14.29	105.03	20.19	20.24	93.86	10.08	11.31	95.11	9.40	10.41	101.40	15.13	15.70
26	BDE-183	59.57	108.60	11.54	11.18	101.10	23.51	24.48	89.53	4.87	5.73	100.61	15.10	15.80	101.56	20.65	21.40
27	Benalaxyl	11.73	101.52	14.48	15.01	101.59	4.49	4.65	95.51	4.74	5.22	100.23	4.63	4.86	99.99	4.04	4.25
28	Bendiocarb	8.45	105.79	7.77	7.73	95.96	4.86	5.33	97.21	5.10	5.52	102.51	5.03	5.16	98.61	2.73	2.91
29	Bendiocarb metabolite (2, 2-dimethylbenzo-1, 3-dioxol-4-ol)	54.51							96.61	12.05	13.13	92.35	5.16	5.89	102.37	18.93	19.47
30	Benfuracarb	52.63				97.77	12.48	13.43	112.31	14.78	13.85	105.31	20.90	20.89	95.61	17.44	19.20
31	Benzo[a]anthracene	23.00	100.95	13.34	13.91	104.58	7.80	7.85	86.86	12.31	14.92	104.46	13.94	14.04	97.66	7.66	8.26
32	Benzo[a]pyrene	18.97	97.86	14.37	15.46	105.41	9.73	9.71	90.28	17.88	20.85	96.05	8.26	9.06	101.85	6.47	6.68
33	Benzo[b]fluoranthene	37.06	107.89	12.46	12.16	100.81	14.27	14.90	91.94	9.57	10.95	91.76	11.46	13.15	105.88	12.46	12.39
34	Benzo[ghi]perylene	39.89	96.46	10.58	11.55	103.87	8.89	9.01	98.58	13.31	14.21	97.68	10.19	10.98	100.50	13.77	14.42
35	Benzo[k]fluoranthene	31.40	99.90	21.36	22.51	107.59	7.48	7.32	85.41	21.81	23.88	93.19	8.18	9.24	104.38	10.62	10.71
36	Bifenthrin	25.99	107.06	8.54	8.40	109.66	6.37	6.11	90.40	11.88	13.84	103.20	9.74	9.94	98.16	8.77	9.41
37	Bitertanol	8.81	101.72	22.45	23.23	106.23	12.39	12.28	90.03	3.62	4.23	99.49	5.66	5.99	101.65	2.84	2.94
38	Boscalid (formerly nicobifen)	47.03	100.63	19.36	20.25	104.11	8.22	8.31	89.07	12.48	14.74	95.12	16.20	17.93	103.61	16.30	16.56
39	Brodifacoum	28.30	84.19	11.76	14.71	103.80	13.59	13.78	94.44	11.45	12.76	103.36	10.95	11.15	98.97	9.67	10.28
40	Bromadiolone	25.02	90.11	18.91	22.09	95.42	11.38	12.56	94.54	4.72	5.25	102.52	12.54	12.87	99.43	8.58	9.09
41	Bromopropylate	39.80	103.98	10.96	11.09	110.51	9.16	8.73	86.70	15.43	18.73	104.76	15.06	15.13	99.66	13.68	14.45
42	Bromuconazole (two isomers)	30.91	106.55	15.23	15.17	108.81	10.70	10.20	87.97	14.45	17.32	96.38	14.51	15.79	103.14	10.58	10.82
43	Bupirimate	10.85	85.46	18.36	22.62	104.46	4.39	4.42	105.85	10.88	10.82	102.49	6.13	6.30	96.40	2.74	2.99
44	Buprofezin	18.10	95.66	3.40	3.74	107.59	7.25	7.10	96.26	5.46	5.97	99.52	5.86	6.20	100.45	6.24	6.54
45	Cadusafos (ebufos)	20.57	87.81	8.36	10.02	117.52	6.95	6.23	108.46	4.48	4.35	104.39	6.46	6.51	93.16	5.09	5.75
46	Carbaryl	11.55	97.61	7.53	8.12	97.44	2.60	2.81	99.40	7.22	7.65	101.88	5.76	5.95	97.76	3.62	3.90
47	Carbendazim (azole)	10.14	101.48	21.84	22.65	102.71	2.20	2.26	97.10	5.65	6.12	103.15	2.87	2.93	97.85	3.13	3.37
48	Carbofuran	9.94	93.67	4.44	4.98	102.64	3.52	3.61	100.79	5.85	6.11	102.98	3.43	3.51	96.87	2.61	2.84
49	Carbofuran-3-hydroxy	10.85				104.10	6.64	6.72	90.76	7.16	8.31	100.99	1.16	1.21	100.99	3.69	3.85
50	Cefuroxima axetil (two isomers)	10.45	107.63	10.02	9.80	97.71	3.85	4.14	92.71	5.91	6.71	102.92	5.68	5.81	99.41	3.57	3.78
51	Chloramphenicol	15.42							88.16	14.23	16.99	98.52	14.68	15.68	101.47	5.25	5.45
52	Chlorantraniliprole	9.55	108.96	15.78	15.25	99.65	6.49	6.86	95.00	6.38	7.06	101.51	3.72	3.85	99.52	3.26	3.45
53	Chlorfenapyr	11.15				96.05	11.01	12.06	104.19	12.01	12.14	95.66	14.47	15.92	101.29	3.76	3.91
54	Chlorfenvinphos	15.13	93.08	16.18	18.30	107.34	2.55	2.50	103.43	4.19	4.26	103.44	6.18	6.29	95.44	4.05	4.47
55	Chlorobenzilate	21.05	97.87	14.12	15.19	108.68	7.88	7.63	98.29	16.88	18.07	95.40	12.29	13.56	101.32	7.24	7.52
56	Chlorophacinone	55.65							94.25	20.33	24.10	105.39	13.83	13.81	100.36	19.20	20.14
57	Chlorpropham	39.94	92.73	11.96	13.57	110.61	9.51	9.05	96.09	13.80	15.12	98.45	14.97	16.00	100.46	13.78	14.44
58	Chlorpyrifos	39.83	92.57	15.38	17.49	105.52	8.87	8.85	92.26	16.56	18.89	96.32	10.16	11.10	103.09	13.77	14.06
59	Chlorpyrifos methyl	9.93	92.64	15.61	17.73	107.15	8.33	8.18	94.06	15.62	17.48	96.37	16.97	18.54	103.83	2.22	2.25
60	Chlorthal dimethyl	41.20	88.50	10.56	12.56	106.05	10.65	10.58	93.19	12.01	13.57	103.42	12.83	13.06	97.54	13.90	15.00
61	Chrysene	12.16				108.26	2.41	2.34	94.17	4.88	5.46	99.74	5.92	6.25	100.65	4.18	4.37
62	Clindamycin	12.54	72.04	14.13	20.64	112.63	14.50	13.56	108.56	7.37	7.14	102.46	5.05	5.19	95.27	2.77	3.06
63	Clofentezine	7.68				99.14	24.98	24.52	94.91	9.26	10.27	101.49	5.13	5.32	99.88	2.64	2.78
64	Clothianidin	10.99				98.28	11.10	11.89	97.34	9.80	10.60	100.77	5.37	5.61	99.70	3.77	3.98
65	Cortiscosterone 21 acetate	22.42				94.83	18.73	20.79	105.17	22.77	22.79	109.51	13.73	13.20	95.93	7.04	7.72
66	Coumachlor	21.24				107.31	11.49	11.27	107.08	7.04	6.92	103.11	6.14	6.27	95.47	6.46	7.12
67	Coumaphos	12.93				95.94	19.86	21.79	93.39	15.06	16.97	101.10	6.93	7.21	99.47	4.42	4.68
68	Coumatetralyl	10.65				100.62	2.32	2.43	102.04	11.53	11.89	102.08	6.66	6.87	97.23	3.08	3.34
69	Cyazofamid	13.81	93.47	12.35	13.91	99.45	5.13	5.43	103.02	6.37	6.51	101.38	5.49	5.70	97.05	4.23	4.59
70	Cyflufenamid	37.37							96.60	10.58	11.53	92.95	11.22	12.71	103.79	12.85	13.03
71	Cyfluthrin (sum of four isomers)	24.66							98.74	12.55	13.37	101.21	6.88	7.16	100.31	8.50	8.92
72	Cyhalothrin (lambda isomer)	10.29				107.25	5.73	5.62	95.42	6.79	7.49	101.83	4.31	4.46	99.67	3.53	3.73
73	Cymoxanil	36.58				105.21	4.95	4.95	94.59	8.18	9.11	98.88	16.00	17.03	101.34	12.65	13.14
74	Cypermethrin (sum of four isomers)	10.29	105.20	7.63	7.64	96.50	5.24	5.71	95.78	8.25	9.07	100.63	6.82	7.13	99.76	3.54	3.73
75	Cyproconazole (two isomers)	15.50				104.49	8.19	8.25	99.15	9.59	10.18	104.91	11.76	11.80	97.82	5.06	5.45
76	Cyprodinil	7.00							92.46	12.18	13.86	100.99	7.51	7.83	99.58	2.39	2.53
77	Cyromazine	42.82							103.52	9.90	10.06	97.41	24.47	24.44	116.05	20.00	18.14
78	Danofloxacin	23.86	75.98	14.99	20.77	117.29	12.71	11.40	93.72	15.08	16.94	105.40	16.03	16.01	97.73	7.97	8.58
79	Dazomet	13.54	95.56	19.38	21.35	103.50	6.07	6.17	95.26	12.19	13.47	99.29	13.17	13.96	100.39	4.66	4.89
80	Deltamethrin	33.46	93.86	20.04	22.47	105.04	15.85	15.88	92.37	16.09	18.34	92.78	13.85	15.71	109.54	9.91	9.52
81	Demeton-S-methyl	47.32				116.16	12.82	11.62	89.95	16.43	19.23	98.03	24.66	24.48	93.53	22.92	23.79
82	Demeton-S-methyl-sulfone (Dioxydemeton)	39.00				111.73	11.57	10.90	90.66	10.87	12.62	100.51	13.73	14.38	100.55	13.46	14.09
83	Dexamethasone	11.06	92.13	8.53	9.75	105.54	7.70	7.68	101.60	6.30	6.53	101.94	5.30	5.47	97.20	3.23	3.50
84	Diazinon	3.06				92.46	6.30	7.18	93.38	3.99	4.50	101.49	5.21	5.41	100.06	1.05	1.11
85	Dibenzo[a,h]anthracene	16.68				112.64	14.38	13.43	90.03	10.00	11.69	97.97	7.56	8.12	101.52	5.69	5.90
86	Dichlorodiphenyldichloroethane (p,p′ DDD)	37.62	95.53	16.47	18.15	107.32	7.25	7.11	89.48	15.34	18.05	90.24	18.81	21.94	106.76	12.50	12.32
87	Dichlorodiphenyldichloroethylene (p,p′ DDE)	34.11	106.07	13.76	13.66	99.57	8.40	8.88	94.20	14.12	15.78	98.30	8.73	9.35	101.23	11.79	12.26
88	Dichlorodiphenyltrichloroethane (p,p′ DDT)	14.94				108.60	4.09	3.97	90.66	14.41	16.73	103.97	14.16	14.34	99.70	5.13	5.42
89	Diclofenac	11.14				105.64	4.06	4.05	93.24	5.48	6.18	99.12	4.97	5.28	101.60	3.70	3.84
90	Dicloran	10.23							100.39	22.88	23.99	105.92	7.34	7.29	98.48	3.34	3.57
91	Diclorvos	38.24				111.23	23.60	22.33	93.47	17.10	19.26	94.62	11.43	12.72	102.13	13.23	13.64
92	Dieldrin	11.43	98.64	14.64	15.62	102.41	3.65	3.75	96.37	6.18	6.75	100.66	6.97	7.29	99.81	3.93	4.14
93	Diethathyl ethyl	14.86	88.24	7.95	9.48	108.70	2.58	2.50	105.93	3.97	3.95	102.90	4.63	4.73	95.31	3.87	4.27
94	Diethofencarb	20.11	102.72	17.08	17.51	101.57	3.86	4.00	99.43	8.58	9.09	102.32	9.12	9.38	97.62	6.64	7.16
95	Difenacoum	12.25	80.96	11.55	15.01	102.72	5.45	5.59	100.39	8.05	8.44	102.92	4.94	5.05	97.20	3.69	4.00
96	Difenoconazole	19.48				102.74	14.93	15.30	101.47	14.00	14.52	103.11	9.15	9.34	96.67	6.19	6.74
97	Difethialone	45.00							101.59	7.31	7.58	94.70	14.77	16.42	103.05	15.60	15.93
98	Difloxacin	19.08				90.37	18.36	21.39	102.44	15.11	23.80	105.09	11.28	11.30	96.33	5.94	6.49
99	Diflubenzuron	25.99	96.88	18.65	20.26	107.54	14.71	14.39	97.24	17.62	22.90	96.41	10.42	11.37	103.53	8.77	8.92
100	Diflufenican	10.75	96.03	3.56	3.91	104.44	1.69	1.70	97.50	5.98	6.45	99.88	5.87	6.18	100.00	3.70	3.90
101	Dimethenamid-P (and its R-isomer)	9.82				102.25	5.36	5.51	95.04	6.05	6.70	101.97	4.95	5.11	99.49	3.36	3.55
102	Dimethoate	12.67	94.43	16.66	18.57	100.31	6.33	6.64	101.63	9.07	9.40	101.57	7.88	8.16	97.78	4.04	4.35
103	Dimethomorph (two isomers)	37.37	89.48	12.23	14.39	111.11	7.12	6.74	93.84	17.37	19.49	95.28	10.47	11.57	103.95	12.83	12.99
104	Dimethylphenylsulfamide (DMSA, metabolite of dichlofluanid)	24.15	111.52	19.40	18.31	99.56	6.10	6.45	89.53	13.95	16.40	94.14	12.44	13.91	104.15	7.98	8.06
105	Diniconazole-M	25.63				98.33	21.68	23.21	105.75	11.68	11.62	102.36	11.88	12.21	96.91	8.42	9.15
106	Dinocap	54.01							106.98	18.75	18.45	90.90	9.62	11.14	104.03	18.77	18.99
107	Diphacinone	30.26	70.85	17.52	19.03	105.76	17.44	17.36	95.74	15.70	17.26	97.12	16.78	18.18	101.09	10.45	10.88
108	Diphenylamine	30.91				101.83	7.15	7.39	96.13	10.34	11.32	101.68	9.00	9.32	99.37	10.60	11.23
109	N,N-dimethylformamidine (DMF, metabolite of amitraz)	26.22							94.68	6.70	7.45	103.33	11.73	11.94	97.94	8.82	9.48
110	Dodine	11.59				110.75	4.01	3.81	93.52	7.93	8.93	100.22	6.75	7.09	100.39	3.99	4.18
111	Endosulfan alfa	17.85				98.84	3.86	4.11	100.61	4.54	4.75	101.95	7.04	7.27	97.48	5.82	6.28
112	Endosulfan beta	8.72				91.54	6.43	7.39	100.39	5.32	5.57	103.11	6.74	6.88	97.25	2.30	2.49
113	Endosulfan sulfate	44.34	102.03	12.59	12.99	106.47	16.24	16.06	91.46	14.51	16.70	98.19	11.21	12.02	102.13	15.37	15.84
114	Endrin	28.51	105.62	11.47	11.43	104.87	14.89	14.94	90.35	12.35	14.39	97.33	10.72	11.59	103.11	9.73	9.94
115	Enrofloxacin	49.49	115.11	10.88	9.95	92.23	10.15	11.58	90.60	16.66	19.36	101.21	23.80	24.76	103.66	24.24	24.62
116	EPN	30.69				118.43	4.97	4.42	93.36	21.86	24.65	96.81	15.31	16.65	106.31	9.94	9.84
117	Epoxiconazole	44.58							98.81	18.22	19.41	96.32	24.41	24.67	103.51	22.51	22.89
118	Eprinomectin	47.23	109.58	10.88	10.45	99.07	6.16	6.54	86.37	12.62	15.38	103.27	17.33	17.67	98.92	16.16	17.19
119	Eritromicin	10.61	112.06	10.02	9.42	95.45	5.64	6.23	92.98	7.30	8.26	101.46	5.40	5.60	99.72	3.64	3.85
120	Esfenvalerate	47.92							99.09	14.55	15.45	94.54	8.79	9.78	91.95	14.85	17.00
121	Ethion (diethion)	17.38	107.84	6.55	6.40	100.92	3.32	3.47	94.93	4.63	5.13	102.77	6.04	6.19	98.59	5.86	6.26
122	Ethirimol	28.11				97.99	14.21	15.26	94.70	16.88	18.77	98.41	12.08	12.92	101.62	9.70	10.05
123	Ethofumesate	16.21	91.25	11.64	13.43	110.47	4.31	4.11	106.91	6.92	6.81	102.24	5.81	5.98	95.56	4.56	5.02
124	Ethoprophos	10.21				101.69	2.29	2.37	92.72	7.46	8.47	102.49	4.21	4.32	99.82	3.51	3.70
125	Etofenprox	53.62	100.20	15.93	16.73	109.55	5.22	5.02	92.07	14.26	16.30	98.97	18.40	19.57	109.18	18.13	17.48
126	Etoxazole	12.52	82.17	11.50	14.74	106.77	4.94	4.87	103.52	6.52	6.63	102.93	5.05	5.17	95.93	3.20	3.52
127	Famoxadone	29.18	85.42	14.45	17.81	115.74	6.23	5.67	94.81	12.30	13.65	98.59	14.01	14.96	100.27	10.06	10.56
128	Fenamidone	34.54	96.20	9.43	10.31	106.75	3.34	3.29	97.38	5.53	5.98	97.70	6.43	6.93	104.34	11.77	11.87
129	Fenamiphos	16.04				103.97	16.68	16.89	98.60	13.26	14.16	103.76	6.17	6.26	96.77	4.97	5.40
130	Fenamiphos sulfone	21.54	79.80	11.11	14.66	104.85	11.91	11.95	104.61	13.51	13.59	102.75	7.74	7.93	95.11	6.43	7.12
131	Fenamiphos sulfoxide	13.37	103.04	14.97	15.29	97.12	7.01	7.60	98.03	12.13	13.02	100.27	6.16	6.46	99.91	4.60	4.85
132	Fenarimol	15.17	107.00	8.52	8.38	102.18	3.35	3.45	93.40	6.06	6.83	100.78	6.32	6.60	100.05	5.23	5.50
133	Fenazaquin	13.05				105.73	16.63	16.56	96.32	6.58	7.19	99.41	5.94	6.29	99.69	4.48	4.73
134	Fenbendazole	42.78	96.23	19.95	21.82	109.39	5.34	5.14	89.90	10.82	12.66	95.35	17.12	18.90	93.89	13.64	15.30
135	Fenbuconazole	19.60	98.06	10.35	11.12	102.23	3.41	3.51	96.79	6.16	6.70	98.96	6.15	6.54	100.65	6.76	7.07
136	Fenbutatin oxide	10.78	97.94	8.11	8.72	99.88	1.64	1.73	103.72	7.34	7.44	103.52	7.08	7.20	95.97	2.41	2.64
137	Fenhexamid	40.07	90.08	8.82	10.30	114.44	14.05	12.92	94.27	11.50	12.84	93.50	13.12	14.77	103.85	13.81	14.00
138	Fenitrothion	40.91	95.76	12.40	13.64	95.28	7.34	8.11	91.70	5.97	6.85	99.29	10.07	10.68	101.66	14.16	14.66
139	Fenoxycarb	12.29				86.84	13.72	16.63	91.58	17.17	19.73	100.48	9.06	9.49	99.83	4.23	4.46
140	Fenpropathrin	35.65				86.56	20.78	24.27	81.25	14.38	18.63	98.13	18.26	19.58	103.85	12.22	12.39
141	Fenpropidin	12.15	95.06	6.49	7.19	106.46	8.00	7.91	105.99	8.98	8.92	102.43	7.54	7.75	95.33	2.61	2.88
142	Fenpropimorph	35.90				103.28	13.33	13.58	90.34	13.75	16.02	103.55	6.98	7.09	97.50	12.08	13.04
143	Fenpyroximate	12.14	95.44	5.24	5.78	101.85	2.69	2.78	102.16	7.02	7.24	101.62	6.06	6.28	97.48	3.75	4.05
144	Fenthion	9.38	90.36	7.35	8.56	98.88	2.96	3.15	97.47	6.22	6.72	102.02	6.88	7.10	97.91	2.86	3.08
145	Fenthion oxon	10.55	84.90	13.69	16.97	103.86	2.51	2.55	104.88	6.93	6.95	103.80	6.09	6.18	95.42	1.75	1.93
146	Fenthion oxon sulfone	20.43	96.71	11.10	12.08	102.96	5.14	5.25	88.90	15.24	18.05	99.53	6.94	7.34	101.39	7.02	7.29
147	Fenthion oxon sulfoxide	9.90	85.66	11.30	13.88	105.85	4.41	4.39	101.77	5.58	5.78	102.66	5.41	5.54	96.46	2.33	2.55
148	Fenthion sulfone	10.05				107.97	6.70	6.53	92.79	4.16	4.71	100.24	5.77	6.06	100.92	3.42	3.57
149	Fenthion sulfoxide	7.50				99.96	4.85	5.10	92.76	6.52	7.39	101.15	5.18	5.39	99.99	2.58	2.72
150	Fenvalerate	18.02				106.51	10.34	10.22	94.20	4.12	4.60	100.52	7.73	8.10	100.12	6.21	6.53
151	Fipronil	10.85				102.94	2.32	2.37	96.57	4.16	4.54	101.76	6.99	7.23	98.21	3.49	3.74
152	Fipronil sulfide	27.97				100.06	11.14	11.72	96.61	12.67	13.81	98.42	12.29	13.14	100.61	9.65	10.10
153	Flocoumafen	32.20	103.31	18.68	19.03	106.30	11.28	11.17	84.22	19.69	24.61	92.29	17.76	20.25	106.01	10.60	10.52
154	Fluazinam	46.45				114.73	14.06	12.90	95.47	20.10	22.16	92.11	16.31	18.64	103.77	16.09	16.32
155	Flubendiamide	24.22	86.88	15.73	19.06	102.45	7.19	7.38	105.55	9.72	9.70	103.52	13.19	13.41	94.98	7.38	8.18
156	Flucythrinate (two isomers)	10.65				100.27	9.51	9.98	96.64	4.82	5.25	100.57	5.45	5.70	100.22	3.67	3.85
157	Fludioxonil	8.71				104.15	20.63	20.85	95.66	13.35	14.69	103.64	8.36	8.49	97.76	2.54	2.74
158	Flufenoxuron	33.79	98.86	17.23	18.38	109.09	6.89	6.63	88.18	11.93	14.25	96.14	14.12	15.49	100.17	11.63	12.25
159	Flumequine	38.28	91.35	16.93	19.51	109.03	17.09	16.50	95.00	11.81	13.08	96.70	9.83	10.70	107.39	12.61	12.36
160	Flunixin	27.62				97.46	6.48	7.00	107.67	9.38	9.17	103.82	9.75	9.89	95.15	8.67	9.59
161	Fluopyram	21.85	105.27	4.00	4.00	96.44	4.45	4.86	102.22	5.46	5.63	103.09	6.89	7.04	97.63	7.25	7.82
162	Fluoranthene	13.72				104.43	8.69	8.76	100.36	3.65	3.83	104.45	4.68	4.72	96.53	4.00	4.36
163	Fluorene	20.70	101.43	23.18	24.05	113.31	18.05	16.77	89.67	14.11	16.57	98.30	14.65	15.69	101.25	7.12	7.41
164	Fluquinconazole	29.95	95.42	20.55	22.67	100.08	8.51	8.95	90.35	13.57	15.81	98.39	14.17	15.16	101.82	10.33	10.68
165	Flusilazole	32.98				126.43	13.07	10.88	97.89	10.72	11.52	97.24	23.61	24.55	100.38	11.38	11.93
166	Flutolanil	60.00	92.66	12.40	14.08	101.18	14.94	15.54	95.13	19.18	21.22	93.62	15.30	17.20	101.91	20.93	21.62
167	Flutriafol	16.79	86.68	17.60	21.37	96.15	16.21	17.75	106.59	16.32	16.11	103.95	5.99	6.07	95.04	4.54	5.03
168	Fluvalinate tau	11.32	92.52	12.02	13.68	106.67	5.42	5.35	96.33	5.22	5.70	100.64	5.53	5.78	99.84	3.89	4.11
169	Fonofos	24.20	90.07	8.01	9.36	108.25	4.63	4.50	94.23	12.72	14.21	98.24	7.48	8.01	101.48	8.34	8.65
170	Formetanate	41.20				99.70	3.01	3.18	93.95	8.37	9.38	96.01	13.07	14.33	101.90	14.27	14.74
171	Fosthiazate	28.65	89.32	16.62	19.59	101.72	6.97	7.22	90.25	10.09	11.77	93.78	16.03	17.99	106.61	9.04	8.93
172	Heptachlor	56.79							98.56	14.43	15.41	104.04	21.87	22.13	99.77	19.53	20.61
173	Hexachlorobencene	10.35	95.58	6.30	6.94	101.59	1.16	1.20	99.57	6.00	6.34	102.20	5.31	5.47	97.65	3.14	3.38
174	Hexachlorocyclohexane (alpha)	29.68	100.23	18.01	18.91	101.88	7.48	7.73	86.97	13.33	16.13	97.06	15.73	17.06	104.25	10.01	10.10
175	Hexachlorocyclohexane (beta)	37.80	87.87	7.48	8.96	105.32	11.04	11.03	96.62	14.46	15.76	103.13	20.13	20.55	108.95	11.97	11.57
176	Hexachlorocyclohexane (delta)	8.40	92.16	12.43	14.19	101.83	9.13	9.44	89.92	14.21	16.64	102.61	14.75	15.13	100.23	2.89	3.04
177	Hexaclorocyclohexane (gamma, lindane)	29.92	100.36	8.08	8.47	103.61	10.52	10.69	96.46	21.78	23.76	99.08	20.02	21.28	102.03	10.31	10.64
178	Hexaconazole (two isomers)	31.46				100.10	10.03	10.55	88.50	11.45	13.62	97.93	18.94	20.36	103.54	10.75	10.93
179	Hexaflumuron	35.88	97.06	9.57	10.38	113.20	23.40	21.76	92.15	10.14	11.58	96.83	10.30	11.20	103.17	12.36	12.61
180	Hexythiazox	15.43				97.80	10.18	10.95	105.35	8.07	8.06	104.96	5.88	5.89	94.59	3.67	4.09
181	Imazalil (enilconazole)	18.45	97.75	14.01	15.09	101.04	5.97	6.22	100.01	7.81	8.22	101.55	6.69	6.94	97.44	6.02	6.50
182	Imidacloprid	7.84	101.35	22.14	22.99	98.35	3.04	3.25	92.25	5.84	6.66	101.60	6.37	6.60	100.02	2.70	2.84
183	Indeno [1,2,3-cd] pyrene	12.29				97.23	11.55	12.50	92.72	10.42	11.83	98.82	4.88	5.20	101.76	4.09	4.24
184	Indoxacarb	32.38	99.75	15.92	16.80	106.45	6.90	6.82	95.30	14.81	16.36	102.04	15.42	15.91	99.49	11.11	11.76
185	Iprodione	26.46				97.52	2.64	2.85	95.46	9.30	10.25	103.31	11.12	11.33	98.00	8.91	9.57
186	Iprovalicarb	48.25				103.44	9.99	10.17	86.34	15.08	18.38	101.33	23.37	24.28	97.77	16.35	17.60
187	Isocarbophos	16.58	81.00	9.98	12.97	111.09	0.64	0.61	107.48	7.55	7.39	104.41	5.94	5.98	93.67	3.56	4.00
188	Isofenphos methyl	26.73	90.40	17.60	20.50	113.42	13.97	12.96	92.51	16.14	18.37	95.85	11.95	13.12	102.59	9.15	9.39
189	Isoprothiolane	10.92	102.55	14.09	14.46	100.61	2.07	2.17	99.19	7.14	7.58	100.34	5.36	5.63	99.87	3.76	3.96
190	Ivermectin B1a	16.57	89.05	14.13	16.70	112.83	15.13	14.12	94.12	10.84	12.12	96.43	7.65	8.35	102.21	5.56	5.73
191	Josamycin	43.99				101.89	20.70	21.39	99.76	11.29	11.91	101.30	11.89	12.35	99.69	15.12	15.97
192	Ketoprofen	15.53	114.79	8.64	7.93	97.82	4.08	4.39	94.19	4.56	5.10	101.27	6.16	6.40	100.54	5.35	5.60
193	Kresoxim methyl	9.41				102.73	18.09	18.53	97.66	8.80	9.49	97.14	6.24	6.76	101.48	3.10	3.21
194	Leptophos	18.10				99.43	13.59	14.39	101.04	16.02	16.69	104.54	5.93	5.97	96.08	5.50	6.03
195	Levamisole	56.89	105.30	19.15	19.14	93.77	8.40	9.43	85.56	10.53	12.95	95.50	15.78	17.39	104.79	19.78	19.87
196	Lincomycin	12.57				93.07	12.69	14.36	99.86	11.37	11.98	98.92	2.42	2.58	100.26	4.33	4.55
197	Linuron	23.14				106.60	13.65	13.48	99.47	6.16	6.52	97.09	13.74	14.90	101.71	7.95	8.23
198	Lufenuron	38.43				96.22	15.91	17.40	94.36	21.11	23.55	93.60	21.32	23.11	105.14	13.09	13.10
199	Malaoxon	9.79				101.17	7.70	8.01	97.98	9.53	10.24	103.11	4.58	4.68	97.64	2.92	3.14
200	Malathion	11.22	82.62	13.11	16.71	100.25	6.92	7.26	105.86	13.54	13.47	102.32	7.46	7.68	95.75	2.46	2.71
201	Mandipropamid	12.89	93.28	5.57	6.29	105.42	0.28	0.28	103.46	5.54	5.63	102.64	4.44	4.55	96.42	3.62	3.95
202	Mebendazole	14.17	101.04	17.64	18.38	103.15	3.49	3.56	96.36	6.42	7.01	100.18	4.99	5.25	99.95	4.88	5.14
203	Mefenamic acid	13.26	90.24	9.15	10.67	108.42	3.34	3.24	101.01	6.31	6.57	103.48	6.31	6.42	95.81	3.46	3.80
204	Mefenoxam (metalaxyl-M)	10.25	103.36	12.88	13.12	102.13	3.34	3.44	96.29	5.54	6.06	101.36	5.62	5.83	99.72	3.52	3.72
205	Meloxicam	27.03				111.41	23.58	22.28	87.66	7.84	9.41	101.76	10.58	10.95	99.94	9.31	9.80
206	Mepanipyrim	13.48	97.45	11.59	12.52	104.03	4.06	4.10	96.00	5.27	5.78	100.55	4.75	4.97	99.83	4.64	4.89
207	Mepiquat	20.05				104.81	8.31	8.34	91.46	8.61	9.90	98.44	4.48	4.79	100.48	6.91	7.24
208	Metaflumizone	34.41				116.29	9.64	8.72	88.22	13.33	15.90	95.73	10.36	11.39	103.80	11.78	11.95
209	Metalaxyl	9.37				102.27	7.69	7.92	92.08	6.45	7.37	100.44	7.75	8.12	100.70	3.20	3.35
210	Metaldehyde	24.07	105.40	9.77	9.76	102.17	7.06	7.27	97.79	4.17	4.49	99.76	4.73	4.99	100.25	8.30	8.71
211	Metconazole	20.49	104.02	15.43	15.62	108.15	9.70	9.44	91.84	10.50	12.03	98.19	11.35	12.16	101.68	7.02	7.27
212	Methamidophos (two isomers)	2.94				110.87	18.67	17.72	90.48	6.12	7.12	100.47	2.55	2.68	100.92	0.80	0.83
213	Methidathion	11.98	97.05	8.53	9.25	103.75	4.62	4.69	99.93	6.66	7.01	98.82	4.46	4.75	100.30	4.13	4.33
214	Methiocarb	15.30							93.85	7.21	8.09	98.11	7.61	8.16	101.60	5.20	5.38
215	Methiocarb-sufone	8.59	95.91	4.45	4.88	101.31	2.62	2.73	94.97	7.05	7.81	100.51	5.19	5.43	99.78	2.95	3.11
216	Methiocarb-sulfoxide	13.23	88.78	5.90	6.99	104.86	6.82	6.84	104.54	6.17	6.21	101.82	5.30	5.48	96.86	3.94	4.28
217	Methomyl	7.40				108.31	5.15	5.01	93.67	7.23	8.12	98.37	5.92	6.33	101.47	2.36	2.45
218	Methoxyfenozide	9.06				101.82	3.15	3.25	91.64	7.20	8.27	102.14	3.70	3.81	100.27	3.12	3.28
219	Metoxychlor	10.18				99.59	3.79	4.00	97.39	6.35	6.86	102.08	3.77	3.89	98.23	3.26	3.49
220	Metrafenone	54.30				109.03	19.34	18.67	86.42	18.34	22.34	101.53	19.54	20.25	105.46	18.82	18.79
221	Metronidazole	11.22	97.19	11.15	12.07	103.48	4.54	4.62	97.54	5.86	6.33	99.29	6.04	6.40	100.33	3.86	4.05
222	Mevinphos (phosdrin)	14.05	88.35	12.61	15.02	114.31	1.58	1.46	104.46	7.37	7.42	103.89	5.30	5.37	95.13	3.47	3.83
223	Mirex	11.87				111.14	9.41	8.91	90.78	6.53	7.57	97.50	7.31	7.89	102.24	3.83	3.94
224	Monocrotophos	6.77				111.72	7.23	6.81	92.90	9.23	10.46	99.61	3.94	4.16	100.46	2.32	2.43
225	Moxidectin	41.02	98.76	13.82	14.73	103.02	14.72	15.04	88.79	12.89	15.28	97.74	17.16	18.48	99.42	14.08	14.91
226	Myclobutanil	6.85				110.18	3.93	3.76	92.89	9.45	10.71	100.33	5.38	5.65	100.68	2.32	2.43
227	N-(2,4-dimethylphenyl)-N’-methylformamidine (DMPF, metabolite of amitraz)	42.13				106.14	14.98	14.85	89.18	9.08	10.72	99.36	14.87	15.75	101.61	14.59	15.11
228	N,N-Dimethyl-N’-p-tolylsulphamide (DMST, metabolite of tolyfluanid)	10.56	97.35	10.02	10.83	104.27	7.42	7.49	98.60	6.49	6.93	98.66	6.22	6.64	100.29	3.64	3.82
229	Naphtalene	18.07				104.69	24.28	24.42	84.22	12.02	15.02	91.04	21.60	24.98	105.57	5.04	5.02
230	Naproxen	18.08							104.76	11.99	12.05	97.40	2.15	2.32	100.44	6.23	6.53
231	Novobiocin	23.78							86.15	21.57	24.36	101.53	6.13	6.35	101.37	8.19	8.51
232	Nuarimol	21.37	95.42	14.07	15.53	114.10	20.37	18.79	96.41	18.11	19.77	97.46	10.74	11.60	101.04	7.36	7.67
233	Ofurace	44.92	99.42	7.14	7.56	104.81	7.72	7.75	90.11	11.50	13.43	100.46	18.07	18.94	102.96	15.57	15.92
234	Omethoate	8.16				104.84	7.41	7.44	91.49	6.43	7.40	98.85	4.19	4.46	102.14	2.42	2.49
235	Oxadixyl	9.41				97.67	5.38	5.80	95.25	8.26	9.12	101.81	5.13	5.30	99.40	3.21	3.39
236	Oxamyl	4.96	109.00	5.35	5.17	99.81	5.00	5.28	94.77	5.40	6.00	102.24	4.17	4.30	99.23	1.62	1.72
237	Oxfendazole	14.43	112.00	9.85	9.26	98.20	2.86	3.06	93.13	4.89	5.53	100.75	7.35	7.68	100.63	4.97	5.19
238	Oxolinic acid	8.60				103.85	4.00	4.05	91.68	5.27	6.06	101.15	4.74	4.93	100.96	2.90	3.02
239	Oxydemeton methyl	22.47				102.56	5.85	6.00	92.13	4.64	5.30	99.17	6.15	6.52	101.58	7.73	8.01
240	Oxyfluorfen	26.60	107.15	18.84	18.51	101.78	18.11	18.73	93.71	22.54	24.32	94.68	10.13	11.27	104.32	8.85	8.93
241	Paclobutrazol	29.94	90.31	18.73	21.83	109.85	19.35	18.55	90.96	14.91	17.25	97.89	11.80	12.68	102.25	10.31	10.62
242	Parathion methyl	19.48	98.41	18.23	19.50	104.65	3.64	3.66	91.06	13.26	15.33	97.15	16.56	17.94	104.95	5.92	5.94
243	PCB 28	30.59	90.80	12.67	14.68	106.28	3.43	3.40	95.75	18.66	20.51	97.36	13.88	15.01	101.58	10.56	10.95
244	PCB 52	49.53	96.76	16.68	18.14	110.07	5.96	5.70	91.27	15.70	18.11	100.93	19.12	19.94	105.86	17.06	16.97
245	PCB 77	32.20	103.38	16.60	16.90	102.27	10.81	11.12	98.20	19.63	21.04	97.88	11.57	12.44	102.22	11.11	11.44
246	PCB 81	52.37	108.02	16.73	16.30	103.90	18.60	18.85	89.56	16.73	19.66	87.59	12.33	14.81	102.26	21.67	22.31
247	PCB 101	42.27	87.04	19.39	23.45	109.17	8.10	7.81	92.00	20.82	23.83	95.80	7.97	8.75	103.43	14.62	14.87
248	PCB 105	27.37	99.26	7.43	7.87	100.33	13.58	14.24	92.72	13.12	14.89	93.12	17.28	19.53	105.20	8.95	8.96
249	PCB 114	24.13	103.71	20.87	21.19	94.46	12.18	13.57	91.10	16.36	18.91	96.99	17.69	19.20	102.77	8.20	8.40
250	PCB 118	28.15	97.19	13.12	14.21	99.86	5.10	5.37	92.76	20.78	23.58	96.30	8.30	9.07	102.29	9.68	9.96
251	PCB 123	31.62	101.08	13.85	14.43	100.49	5.75	6.02	95.08	19.70	21.81	94.75	14.61	16.24	102.61	10.88	11.16
252	PCB 126	19.28	96.17	18.84	20.62	107.21	20.06	19.70	94.36	17.42	19.43	96.66	12.75	13.88	102.60	6.48	6.65
253	PCB 138	26.55	96.29	8.95	9.78	100.71	15.36	16.05	93.60	17.01	19.12	97.66	12.37	13.33	100.79	9.16	9.57
254	PCB 153	33.56	92.09	6.60	7.55	101.91	15.13	15.63	95.55	16.13	17.76	98.44	15.61	16.69	101.60	11.60	12.02
255	PCB 156	55.52	89.30	5.79	6.82	104.11	17.82	18.02	92.53	17.92	20.38	101.92	21.37	22.07	99.35	18.94	20.07
256	PCB 157	26.85	95.13	17.25	19.09	104.76	13.27	13.33	89.85	15.11	17.71	102.60	16.72	17.16	99.44	9.21	9.75
257	PCB 167	51.81	113.98	21.88	20.21	99.57	17.14	18.12	87.57	13.30	15.99	94.35	18.40	20.53	93.21	16.57	18.72
258	PCB 169	52.09	101.60	18.21	18.86	103.24	8.55	8.72	85.44	9.25	11.40	90.15	20.58	24.03	97.16	17.56	19.02
259	PCB 180	13.23	104.96	14.37	14.41	106.93	9.30	9.15	88.67	14.22	16.88	93.69	14.56	16.36	100.90	4.53	4.73
260	PCB 189	25.16	103.03	10.15	10.37	100.28	19.19	20.14	89.16	12.30	14.52	93.67	9.90	11.13	104.12	8.36	8.45
261	Penconazole	34.16	95.70	12.80	14.08	115.85	4.50	4.09	95.53	13.40	14.76	93.08	11.71	13.24	99.44	11.72	12.41
262	Pencycuron	20.48	98.16	9.03	9.69	105.55	4.45	4.43	96.26	4.83	5.28	100.53	7.15	7.49	100.12	7.06	7.42
263	Pendimethalin	17.70	90.91	14.47	16.76	105.21	7.43	7.43	99.02	3.67	3.90	102.02	5.04	5.20	97.75	5.82	6.27
264	Penicilina V	26.71							96.64	5.84	6.36	97.01	8.57	9.30	101.26	9.22	9.58
265	Permethrin	36.34				105.69	14.94	14.88	95.27	21.90	24.20	90.29	19.06	22.22	97.91	12.29	13.21
266	Phenanthrene	17.45	98.02	12.31	13.22	99.55	12.44	13.16	93.48	13.10	14.75	93.74	12.82	14.39	103.69	5.55	5.63
267	Phenylbutazone	49.13							91.74	15.73	18.05	93.57	19.01	21.39	105.65	16.94	16.87
268	Phosalone	11.29	100.31	7.80	8.18	102.56	5.32	5.47	94.92	4.35	4.82	99.26	7.11	7.54	100.09	3.89	4.09
269	Phosmet	13.71				104.01	7.48	7.57	98.61	6.23	6.65	103.07	4.47	4.56	97.45	4.32	4.67
270	Phosmet-oxon	7.06				97.85	4.69	5.05	97.35	8.44	9.13	102.67	5.14	5.27	98.02	1.99	2.13
271	Pirimicarb	10.35	92.13	7.98	9.12	108.26	1.33	1.29	103.45	4.95	5.04	103.13	3.94	4.02	95.99	2.20	2.42
272	Pirimicarb-desmethyl	8.59	102.89	7.11	7.27	101.86	3.08	3.18	96.80	6.76	7.35	101.82	3.15	3.26	98.62	2.78	2.97
273	Pirimiphos ethyl	49.06	92.82	19.20	21.77	118.12	2.68	2.39	87.29	11.06	13.34	96.72	14.11	15.36	110.86	15.95	15.15
274	Pirimiphos methyl	46.53	97.86	11.37	12.23	106.89	1.77	1.74	91.79	11.32	12.99	101.94	17.14	17.69	107.79	15.68	15.32
275	Prochloraz	11.75	93.09	13.04	14.74	109.38	9.57	9.21	104.39	6.98	7.03	103.14	5.34	5.45	95.40	2.45	2.71
276	Procymidone	47.71				106.77	7.90	7.79	88.94	18.04	21.35	94.09	11.30	12.65	96.19	15.88	17.38
277	Profenofos	19.09	100.50	11.04	11.56	101.67	8.80	9.11	100.12	6.04	6.35	101.11	7.81	8.13	98.72	6.47	6.90
278	Propamocarb	5.99				103.07	4.64	4.74	87.68	3.95	4.75	100.88	3.74	3.90	101.51	1.80	1.86
279	Propargite	20.43	98.90	9.92	10.56	102.07	5.44	5.61	97.64	5.69	6.14	98.89	6.67	7.10	100.75	7.04	7.36
280	Propiconazole	12.75				96.84	8.80	9.57	100.37	8.04	8.43	100.50	5.91	6.19	98.02	4.13	4.43
281	Propoxur	7.88	106.77	12.22	12.05	100.74	5.19	5.42	95.66	6.74	7.42	102.83	4.63	4.74	99.26	2.65	2.81
282	Propyzamide (pronamide)	7.08	92.65	19.33	21.96	95.89	6.00	6.59	95.61	6.94	7.65	100.37	5.71	5.98	99.85	2.43	2.57
283	Proquinazid	20.15	93.83	12.24	13.73	108.78	6.04	5.85	101.24	7.90	8.22	102.34	6.01	6.18	96.68	6.44	7.01
284	Prothioconazol	20.98	99.10	11.03	11.71	111.77	11.21	10.56	101.24	15.30	15.91	101.44	7.09	7.36	100.39	7.23	7.58
285	Prothiophos	54.52	99.76	20.17	21.29	115.45	19.52	17.79	90.43	16.79	19.54	92.15	10.78	12.31	106.16	18.85	18.69
286	Pymetrozine	12.11				104.52	9.45	9.52	99.96	6.47	6.81	98.85	7.40	7.88	100.89	4.14	4.32
287	Pyraclostrobin	14.46	92.85	6.84	7.76	103.95	2.37	2.40	97.43	5.66	6.11	100.22	6.57	6.90	100.04	4.98	5.24
288	Pyrazophos	13.83	97.27	7.18	7.77	104.11	8.90	9.00	100.36	6.58	6.90	100.04	5.66	5.96	99.70	4.75	5.02
289	Pyrene	35.48	84.48	4.35	5.42	118.27	15.20	13.53	90.78	10.35	12.00	95.73	8.21	9.03	102.81	12.24	12.53
290	Pyridaben	21.92	99.30	12.16	12.89	102.10	3.57	3.68	97.39	5.95	6.43	99.28	7.39	7.83	100.30	7.56	7.93
291	Pyridaphenthion	13.23	100.15	9.50	9.99	101.73	5.42	5.61	96.49	3.96	4.32	100.26	4.92	5.17	99.95	4.55	4.80
292	Pyrimethanil	21.20	95.66	16.08	17.69	105.17	12.00	12.01	91.20	11.13	12.84	94.12	12.46	13.94	103.96	6.91	6.99
293	Pyriproxifen	17.64	96.87	4.83	5.25	105.33	2.02	2.02	98.71	5.79	6.18	97.28	5.56	6.01	91.30	0.94	1.09
294	Quinalfos	11.63	94.48	10.56	11.76	96.90	6.47	7.03	106.43	6.56	6.49	101.72	6.64	6.87	96.67	3.22	3.51
295	Quinoxyfen	17.58	90.90	11.93	13.81	100.16	8.47	8.90	102.02	7.55	7.79	102.26	5.09	5.24	96.99	5.60	6.07
296	Rifampicin	38.32							95.11	8.51	9.42	99.82	8.14	8.58	99.31	13.14	13.93
297	Rotenone	38.71				116.09	14.12	12.80	101.14	19.83	20.64	103.06	11.26	11.50	95.90	12.74	13.98
298	Roxithromycin	16.85				105.39	12.55	12.54	90.66	7.64	8.87	96.26	3.89	4.25	103.64	5.33	5.41
299	Simazine	5.25	106.22	13.48	13.35	95.00	4.14	4.59	93.84	6.29	7.06	101.60	4.45	4.61	99.67	1.79	1.89
300	Spinosad (two isomers)	22.35				105.88	7.75	7.70	106.76	13.25	13.05	100.51	6.11	6.45	97.07	7.36	7.88
301	Spiramycin (two isomers)	6.56							95.12	6.27	6.94	98.30	4.51	4.83	101.25	2.10	2.19
302	Spirodiclofen	15.03							105.95	10.56	10.49	96.95	4.55	4.94	100.27	5.18	5.44
303	Spiromesifen	12.81				101.56	4.83	5.00	99.43	6.18	6.54	97.60	5.75	6.20	100.24	4.41	4.63
304	Spiroxamine	11.53	84.84	7.85	9.74	105.10	1.89	1.90	104.57	7.11	7.15	102.72	4.87	4.99	96.16	2.90	3.17
305	Strychnine	11.90				98.61	6.60	7.04	92.65	8.23	9.35	101.86	5.04	5.21	100.73	4.08	4.27
306	Sulfacetamide	18.66							95.38	12.81	14.13	101.54	7.67	7.95	100.85	6.43	6.71
307	Sulfachloropiridacine	4.66				96.98	0.49	0.53	92.42	5.44	6.19	99.53	3.02	3.20	100.56	1.56	1.64
308	Sulfadiacine	22.38				108.52	15.90	15.42	91.45	5.34	6.15	97.90	5.89	6.33	102.45	7.61	7.82
309	Sulfadimetoxine	10.71	110.25	12.55	11.99	96.34	4.79	5.24	96.18	5.92	6.47	100.38	6.04	6.34	100.47	3.68	3.86
310	Sulfadoxine	7.31	107.49	7.96	7.79	94.89	5.76	6.39	94.43	6.42	7.16	102.25	5.28	5.44	99.73	2.51	2.64
311	Sulfameracine	6.60				103.87	2.73	2.77	94.75	4.37	4.85	100.53	7.96	8.33	100.38	2.26	2.37
312	Sulfametacine	8.13				105.16	1.02	1.02	92.79	8.30	9.42	100.18	5.58	5.86	100.61	2.78	2.90
313	Sulfametizole	4.48				105.55	4.45	4.44	94.95	7.79	8.64	102.75	5.57	5.71	99.45	1.49	1.58
314	Sulfametoxazole	9.86				105.88	3.94	3.92	93.12	8.76	9.90	103.21	4.47	4.56	98.89	3.29	3.50
315	Sulfametoxipiridacine	10.11				103.07	3.90	3.99	89.72	6.58	7.72	101.01	5.53	5.76	100.77	3.46	3.61
316	Sulfamonomethoxine	30.69				120.10	11.58	10.15	93.43	9.85	11.10	100.65	5.38	5.63	100.77	10.60	11.07
317	Sulfapyridine	13.16				107.39	7.54	7.39	95.05	7.02	7.77	98.88	7.30	7.77	101.09	4.49	4.68
318	Sulfaquinoxaline	19.01				108.10	8.28	8.06	92.03	6.07	6.95	98.12	7.23	7.76	102.11	6.45	6.65
319	Sulfatiazole	13.98				107.25	18.23	17.89	91.67	8.92	10.24	100.18	10.87	11.42	100.13	4.82	5.06
320	Sulfisoxazole	6.76				95.64	5.52	6.08	90.14	5.37	6.27	101.96	3.79	3.91	99.82	2.32	2.45
321	Tebuconazole	4.02				96.70	9.65	10.50	96.71	8.87	9.65	100.97	5.13	5.35	99.07	1.22	1.30
322	Tebufenocide	9.66	98.70	9.29	9.90	103.48	4.98	5.07	100.37	9.81	10.29	98.74	6.14	6.55	100.66	3.31	3.46
323	Tebufenpyrad	54.25	98.40	19.31	20.65	103.42	14.26	14.51	87.22	16.18	19.53	96.43	16.30	17.80	93.97	17.61	19.73
324	Teflubenzuron	14.59				103.61	16.19	16.45	91.96	11.61	13.29	99.92	10.40	10.96	100.04	5.03	5.29
325	Tefluthrin	18.98	94.50	18.42	20.52	109.30	5.13	4.94	95.83	13.31	14.62	97.15	10.26	11.12	103.00	6.29	6.43
326	Telodrin (isobenzan)	31.11	93.25	19.35	21.84	116.61	15.64	14.12	89.63	17.21	20.21	94.46	10.00	11.14	104.86	10.43	10.47
327	Terbufos	17.89	89.87	18.10	21.20	101.84	16.58	17.14	98.77	16.02	17.07	99.46	14.72	15.58	105.00	5.24	5.25
328	Terbuthylazine	14.43	93.29	4.25	4.80	109.08	4.95	4.78	96.04	4.63	5.07	98.95	5.77	6.14	100.31	4.97	5.22
329	Tetrachlorvinphos	15.02	86.68	16.44	19.97	104.08	5.78	5.84	103.29	9.75	9.93	100.97	4.69	4.89	96.47	4.48	4.89
330	Tetraconazole	38.67	89.01	16.15	19.10	114.33	10.30	9.48	94.97	19.82	21.97	99.62	11.36	12.01	106.33	12.98	12.85
331	Tetradifon	21.58	92.25	17.26	19.70	108.82	9.05	8.75	97.26	10.04	10.87	98.91	10.35	11.02	99.23	7.38	7.83
332	Tetramethrin	38.36				110.19	8.77	8.38	87.06	11.18	13.51	99.88	13.15	13.85	96.19	12.68	13.88
333	Thiabendazole	6.96	109.34	8.89	8.56	99.89	4.64	4.89	92.53	7.57	8.61	102.95	3.30	3.37	99.44	2.36	2.50
334	Thiacloprid	7.62	100.27	9.33	9.79	99.72	2.71	2.86	96.75	5.67	6.17	102.32	4.32	4.44	98.86	2.49	2.65
335	Thiamethoxam	8.90							94.30	9.01	10.05	101.19	2.45	2.55	99.73	3.06	3.23
336	Thiophanate methyl	9.82	94.93	7.65	8.49	102.70	1.97	2.02	100.03	5.00	5.27	101.78	5.03	5.20	97.82	3.00	3.22
337	Tolclofos methyl	18.85							96.49	2.19	2.39	93.05	7.46	8.44	104.27	5.91	5.97
338	Tolfenamic acid	15.70	96.60	20.72	22.58	113.09	5.70	5.31	93.69	11.93	13.40	95.77	17.20	18.90	101.95	5.29	5.46
339	Triadimefon	15.86				103.69	22.53	24.92	96.37	21.63	23.62	97.25	7.36	7.97	100.42	5.47	5.73
340	Triadimenol	11.21	90.21	16.68	19.46	108.01	10.30	10.04	104.18	2.94	2.97	102.84	6.90	7.07	95.94	2.60	2.86
341	Triazophos (hostathion)	11.28				107.10	15.08	14.83	96.40	6.06	6.62	100.08	4.21	4.43	99.92	3.88	4.09
342	Trichlorfon	11.09	97.09	8.26	8.96	103.21	3.75	3.82	97.44	5.31	5.74	99.08	5.57	5.92	100.42	3.82	4.00
343	Trifloxystrobin	7.09				94.36	14.78	16.49	96.83	5.76	6.26	98.87	4.98	5.31	100.65	2.41	2.52
344	Triflumizole	14.95	98.38	11.61	12.43	99.91	3.85	4.05	97.81	6.89	7.41	99.87	5.89	6.21	100.10	5.15	5.42
345	Triflumuron	10.52	97.57	4.93	5.32	100.94	4.59	4.79	97.95	7.98	8.58	99.43	5.30	5.62	100.20	3.62	3.81
346	Trifluralin	18.05				94.40	16.87	18.82	95.90	6.43	7.05	103.36	7.85	8.00	97.05	5.78	6.27
347	Trimethoprim	47.12				112.07	12.04	11.31	96.24	11.78	12.88	101.74	20.93	21.65	108.42	15.79	15.33
348	Triticonazole	10.27				105.14	4.25	4.26	95.26	6.47	7.15	96.56	7.30	7.96	102.90	2.96	3.03
349	Tylmicosin	10.97				113.79	16.19	14.98	88.65	6.89	8.18	101.93	6.09	6.29	99.54	3.75	3.97
350	Tylosin	10.56				107.72	11.23	10.98	96.69	5.96	6.49	99.51	5.98	6.32	100.64	3.62	3.79
351	Vinclozolin	18.23	87.26	12.21	14.73	113.36	11.73	10.89	98.12	18.88	20.25	96.44	12.88	14.06	102.62	6.09	6.25
352	Warfarin	10.77	87.78	17.57	21.07	91.37	23.07	24.58	95.23	6.45	7.13	102.69	4.32	4.43	98.14	3.45	3.70
353	Zoxamide	17.19	80.27	12.78	16.76	100.65	7.00	7.33	106.21	9.24	9.16	103.64	7.51	7.62	94.40	4.35	4.85
Blank cells were set for compounds that have a higher LOQ than the lowest concentrations tested.																	

## Appendix B. Detected Concentrations (Minimum (Min), Maximum (MAX) and Median (Me) Malues, with Frequencies of Detection) of Analytes in Human Series (*n* = 25)

POPs	min–MAX (ng/mL)	Me (ng/mL)	*n* (%) Positives
DDE-p,p′	0.36–51.4	3.75	23 (92%)
Naphthalene	1.17–3.35	1.62	20 (80%)
HCB	0.3–4.74	0.99	12 (48%)
HCH-B	0.29–4.09	1.34	12 (48%)
PCB-153	0.31–1.14	0.48	10 (40%)
PCB-180	0.32–0.92	0.47	10 (40%)
PCB-138	0.3–0.82	0.44	8 (32%)
Pharmaceuticals	min–MAX (ng/mL)	Me (ng/mL)	*n* (%) Positives
Acetaminophen	4–539.64	16.65	9 (36%)
Naproxen	30.46–4058.04	650.33	3 (12%)
Ketoprofen	7.41–30.93	16.87	3 (12%)
Levamisole	11.98–16.37	14.18	2 (8%)
POP (persistent organic pollutant); DDE-p,p′ (dichlorodiphenyldichloroethylene); HCB (hexachlorobenzene); HCH-B (beta-hexachlorocyclohexane); and PCB (polychlorinated biphenyl).			
